# Macropsychology: A Systematic Scoping Review of the Psychology Literature on Public Policy and Law

**DOI:** 10.3390/bs15030350

**Published:** 2025-03-12

**Authors:** Moonika Moonveld, Joanne McVeigh

**Affiliations:** 1 Department of Psychology, Maynooth University, W23 F2H6 Maynooth, Ireland; 2 Assisting Living & Learning (ALL) Institute, Maynooth University, W23 F2H6 Maynooth, Ireland; joanne.mcveigh@mu.ie

**Keywords:** macropsychology, psychological governance, law, policy, social justice

## Abstract

Macropsychology examines the influence of macro-level factors such as policies and laws on our psychological well-being and how the field of psychology can be more effectively leveraged to influence them. While psychology has traditionally been focused at the individual level, a greater focus is needed on policies and laws at the macro level, including areas that are underpinned by psychological concerns such as human rights and social justice. Systematic scoping review methods based on the PRISMA guidelines were used to examine the following research question: To what extent is psychology, through macropsychology, engaging with public policy and law, particularly in relation to social justice? In total, 118 articles were identified as meeting the inclusion criteria, including 46 empirical articles and 72 conceptual articles. Although the authors of such articles are clearly operating at the macro level, it is not evident that they conceptualise such work as macropsychology. This scoping review is the first to systematically synthesise psychological research at the macro level, adding value to the existing conceptualisation of macropsychology. This review calls attention to the work of psychologists engaging with public policy and law from a social justice perspective.

## 1. Introduction

Psychology has long been focused at the micro and meso levels, less readily examining the impact of policies and law on our psychological well-being and how psychology can in turn be wielded to influence them. While psychology has traditionally been focused at the individual level, a greater focus is needed on policies and law at the macro level, including areas that are underpinned by psychological concerns such as the distribution of resources, power relations, and the settings and conditions required for people to exercise their rights (McVeigh & MacLachlan, 2022). Embracing this macro perspective would allow psychology to have a greater impact with its research findings, to use big data more effectively, and to have more involvement in areas such as organisational and social justice, human rights, equality, and equity (MacLachlan & McVeigh, 2021).

Policies are population-level interventions with the aim of influencing behaviours and choices to produce desired outcomes (Ruggeri, 2017), and the goal of evidence-based policy is to apply scientific research to decision-making (McKnight et al., 2005). The social sciences are therefore intrinsically in the realm of policy and governance (McVeigh & MacLachlan, 2021). Psychologists can valuably contribute to policy content and policy processes, for example, by providing robust models and methods for analysing populations, for supporting sustainable and community-based solutions, and for strengthening decision-making processes (Bullock et al., 2023).

However, the influence of psychological science in the public sphere has historically been relatively limited (Tropp, 2023), although psychology is now increasingly influencing how states operate (Jones & Whitehead, 2018). There has been a proliferation globally of ‘behavioural insights’ projects and units, comprising insights from psychology, cognitive science, and broader social science, schematically presented in the OECD’s Observatory of Public Sector Innovation’s interactive map (see https://oecd-opsi.org/bi-units, accessed on 26 February 2025) (OPSI, 2021).

The application of insights from the psychological sciences is the domain of ‘psychological governance’. Psychological governance may be defined as “forms of largely state-orchestrated public policy activity (though ‘nonstate’ actors are widely involved) that aim to shape the behavior of individuals, social groups, or whole populations through the deployment of the insights of behavioral and psychological sciences” (Pykett et al., 2017, p. 2). Psychological governance therefore focuses on the ways in which people behave, the reasons for such behaviours, and how to influence these behaviours (McVeigh & MacLachlan, 2021).

Psychological governance is facilitated by a macropsychology perspective, which may be defined as “the application of psychology to factors that influence the settings and conditions of our lives” (MacLachlan, 2014, p. 851). Macropsychology addresses how our well-being is influenced by macro-level factors including policies, laws, institutions, systems, and structures. It also examines how the field of psychology can be more effectively leveraged to influence them, whereby psychology needs to go further, beyond advocacy, to focus on evidence-based activism (Carr & MacLachlan, 2014). Various fields in psychology adopt this macro focus, including social psychology, political psychology, and human rights psychology. However, this macro perspective has been predominantly neglected in psychology in favour of reductionist and individualist perspectives (McVeigh & MacLachlan, 2022).

### Research Aim

While scoping reviews have been conducted in cognate areas such as behavioural public policy (Mukhtarov, 2022), behavioural economics (Andrawis et al., 2022), nudging (Van Deun et al., 2018), and choice architecture interventions (Forberger et al., 2019), there is a paucity of reviews of the psychology literature on policy and law. In response to this research gap, a scoping review was conducted of the psychology literature on the engagement of psychology with public policy and law, particularly in relation to social justice.

A particular focus was adopted in the review on social justice due to the importance of law and policies that are rights-based and well rooted in the principles of fairness and social inclusion. As emphasised by the UN, the test of ‘good’ governance is the extent to which it promotes and protects human rights, including economic, social, political, cultural, and civil rights (OHCHR, 2022). Social justice may be defined as fairness, equity, and equality in access to resources, human rights, opportunities, and freedoms (Watts & Hodgson, 2019). It denotes the application of justice principles throughout society such as distributive justice, interactional justice, and procedural justice (Knapp & Fingerhut, 2024). Manifest injustices occur when reasonable means could be used to alleviate disproportionate morbidity and to prevent mortality (Venkatapuram et al., 2010). Evidence-based policies are therefore required that are drafted in the spirit of social justice (Marmot, 2017). Accordingly, psychology must zoom out from the individual level to also examine the systems, institutions, and policies that perpetuate marginalisation and unfairness; otherwise, it risks focusing on the symptoms of social injustice without addressing the root causes and may further marginalise structurally vulnerable groups (MacLachlan, 2017).

Similarly, in recognising law as a social construct rather than a scientific enterprise (Focarelli, 2012), it is important to evaluate the extent to which law, as a psychological instrument to wield behaviour, is rights-based and underpinned by social justice. As proposed by Newman and Gordon (2021),

“[T]he law is not an inanimate rule book for some inherently fair or meritocratic game of individual chance, skill, or even ‘justice’. It can be a powerful engine for the progressive advancement of some or all people or the means of their repression. It is made by humans and so is never completely neutral. It has moral content and values, not only in its substance but in its linguistic framing, form, process, and priorities”.

A scoping review was therefore conducted of the psychology literature on the extent to which psychology, through macropsychology, engages with public policy and law, particularly in relation to social justice. Accordingly, the review examined the areas in which psychology may inform and influence policy and law at the structural level. In doing so, it is hoped that this review calls attention to the work of psychologists engaging with public policy and law from a social justice perspective.

## 2. Methods

Scoping reviews are appropriate for research areas that have not been extensively reviewed, whereby such reviews can be used as a preliminary assessment of the extent, scope, and nature of the literature in a particular area, alongside gaps in the literature (Premachandra & Lewis, 2022). Scoping reviews fulfil various purposes, including identifying the types of evidence in a particular research area, elucidating key concepts in the literature, exploring how research is carried out in a particular field, detecting knowledge gaps, and providing the foundation for a systematic review (Munn et al., 2018).

Accordingly, due to a gap in the literature regarding a review of psychology’s involvement in public policy and law, systematic scoping review methods were conducted to examine the scope and nature of work in this area. In accordance with the framework provided by Arksey and O’Malley (2005), the scoping review was carried out using a methodologically rigorous and transparent approach to allow replicability and to strengthen the reliability of the findings.

### 2.1. Search Strategy

The overarching research question explored in the review was as follows: To what extent is psychology, through macropsychology, engaging with public policy and law, particularly in relation to social justice? Numerous preliminary sensitive search strategies were first developed. A Search Librarian was consulted to discuss the preliminary search strategies and systematic/scoping review methods more broadly. Due to the large number of articles returned from these searches, the search strategy was then revised to a more refined strategy.

**Figure 1:**
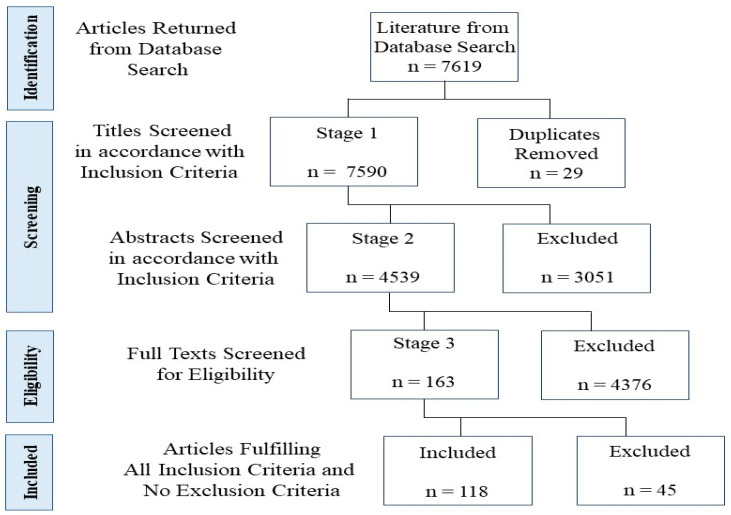
Flowchart of the screening process (in accordance with PRISMA).

The final literature search was conducted on 13 June 2022. The review was not prospectively registered. The inclusion and exclusion criteria are provided in Table 1. The search was conducted on APA PsycInfo using EBSCOHost. A search was conducted for publications on psychology at the macro level, with a particular focus on social justice. A second search was conducted to supplement the main search, specifically using the terms ‘behavioural insights’, ‘behavioural governance’, and ‘behavioural government’. Articles published in peer-reviewed psychology journals were included. The scoping review was conducted in accordance with the PRISMA criteria (please see Figure 1 and Supplementary File S1).

The time scale of 2010 onwards was selected, as the world’s first governmental institution for the application of behavioural sciences in policy, the UK Behavioural Insights Team, was established in 2010 (Hallsworth et al., 2018). The search was conducted in English. However, a number of non-English articles were also returned during the search, as they had an English title and abstract (two languages). Those non-English articles that fulfilled the criteria after abstract screening were translated into English using Google Translate. With regard to papers returned from multidisciplinary journals, articles were included if the first author was working in the field of psychology, as determined by author affiliation. Table 2 presents the search terms used for the review. More details on the electronic search strategy conducted in APA PsycINFO are presented in Supplementary File S3.

### 2.2. Literature Screening

The review was conducted using Rayyan (www.rayyan.ai), an online tool for systematic literature reviews. The primary researcher (MM) reviewed the titles, abstracts, and full texts in accordance with the inclusion criteria. The second reviewer (JMV) screened one in ten articles at the title, abstract, and full-text screening stages. When a disagreement between reviewers arose, this was resolved upon discussion.

### 2.3. Data Extraction

Following the full-text screening stage, both reviewers extracted information from the articles. A data extraction template was designed to record relevant data from the included publications, including the authors, aims, design, population of interest, geographical location, main findings, main conclusions, and key words/topics (see Supplementary File S2). In the extraction template, articles were also categorised as empirical articles or conceptual articles.

## 3. Results

The search strategy yielded 7619 articles, after which 29 duplicates were removed. After title, abstract, and full-text screening were conducted, 118 articles were identified as meeting all of the inclusion criteria and none of the exclusion criteria. Figure 1 schematically presents the scoping review search process. Supplementary File S2 presents the completed data extraction template for all 118 articles, including 46 empirical articles and 72 conceptual articles.

### 3.1. Populations

The articles focused on various populations, as summarised in Table 3 and discussed in more detail below.

#### 3.1.1. Children and Adolescents

In total, 19 articles (16.1%) focused on children and/or adolescents. Such articles examined specific populations such as left-behind children from rural primary schools in remote areas of western China (Article 11) (Please note that article numbers correspond to those presented in Supplementary File S2), children with special healthcare needs (Article 37), youth with obesity (Article 115), families with parental mental health issues and child protection concerns (Article 84), and legal professionals working with lawsuits involving children (Article 44). An overview of the populations that were in focus in the articles is provided in Supplementary File S2.

For example, in a policy analysis of Child and Adolescent Mental Health Services Policy in England, Callaghan et al. (2017) identified how distress experienced by children is individualised via medicalising discourses and understandings of the relationship between socioeconomic circumstances and mental health. In a qualitative study of urban American Indian youth and families, West et al. (2012) found that system/policy changes are needed to support systems of care that address the needs of communities. Magor-Blatch (2011) critically examines the ‘Zero Tolerance’ policy, arguing that a better understanding of adolescent development and working more proactively within a harm minimisation and primary prevention model will provide better opportunities for at-risk children and young people.

With regard to inclusive education policy, Thomas (2013) argues that inclusive education needs to engage with a different psychology of learning, a model of learning that emphasises the significance of community including meaning, narrative, and apprenticeship (i.e., the context and culture for learning). In a mixed methods study on child and adolescent mental health policies, programmes, and infrastructures across Europe, Bielsa et al. (2010) reported inter alia that the implementation and effects of child and adolescent mental health (CAMH) policies and action plans are not evaluated systematically in Europe.

#### 3.1.2. People with Mental Health Problems

A number of articles focused on people with mental health problems and/or substance abuse problems and/or service users more broadly (16, 13.6%) (Articles 19, 22, 23, 27, 77, 88, and 90); individuals found not criminally responsible on account of a mental disorder (Article 79); mental health service users and people subject to (involuntary) detention (Articles 1 and 10); mental health system stakeholders including service users and advocates more broadly (Articles 6, 36, 47, and 51); people with mental, neurological, and substance use disorders in low- and middle-income countries (Article 27), and populations with lived experience in the Movement for Global Mental Health (Article 107).

For example, Cronin et al. (2017) conducted a narrative review of mental health legislation across the Republic of Ireland, England and Wales, Scotland, Ontario (Canada), and Victoria (Australia). While they found broadly similar procedures across the five jurisdictions for the admission, detention, and treatment of involuntary patients, they identified differences with regard to the criteria in defining a ’mental disorder’, automatic review hearings subsequent to a patient being involuntarily admitted, and supported decision-making under mental health legislation. Kitafuna (2022) presents an overview of mental health-related beliefs, services, and systems in Uganda and recent activist and legal challenges. As contended by Kitafuna, while Ugandans with mental health problems experience significant stigma, social exclusion and punitive treatment, incremental improvements and revolutionary advancements have been evident in the last 2–3 decades as a result of momentum by activists with psychosocial disabilities, in addition to the UNCRPD, international disability alliances, and international law.

Wong et al. (2014) critically explored Chinese mental health and mental health-related policies, raising numerous policy-related questions such as the inadequate coverage of mental health services in rural districts and inadequate protection of the rights of individuals with mental health problems. In a critical review of the Lancet Commission on global mental health and sustainable development, Cosgrove et al. (2020a) called for a paradigm shift that adopts a ‘global burden of obstacles’ approach (one that addresses structural issues), which aligns with a politically informed societal determinants of health framework. Accordingly, instead of conceptualising poverty, violence, or gender inequity as predictive variables or risk factors for mental health problems, Cosgrove et al. (2020a) argued that we need to view the crisis in mental health as a crisis of obstacles. In a qualitative study exploring challenges in the South African mental health system, Lund et al. (2011) identified key challenges as including the lack of officially endorsed mental health policy, the low priority of mental health, and inadequate intersectoral policy integration.

#### 3.1.3. LGBTQ+ Persons

Several articles focused on LGBTQ+ persons (13, 11%), including individuals in same-sex relationships (Article 4); Lesbian, Gay, and Bisexual (LGB) individuals (Articles 32 and 104); LGBTQ+ individuals (Articles 13, 62, and 87); LGBTQ+ service users and professional practitioners such as psychologists (Articles 34 and 103); sexual minority (LGBQ+) college students (Article 20); sexual and gender minority individuals (Article 9); transgender and gender-diverse (TGD) individuals (Articles 17 and 99); and parents of trans and gender-diverse (TGD) youth (Article 15).

For example, Ogolsky et al. (2019) explored changes in personal well-being among people in same-sex relationships throughout the transition to federal marriage recognition, using longitudinal panel data collected before and after the US Supreme Court decision in *Obergefell v*. *Hodges*. The findings indicated that the Supreme Court decision, which provided legal recognition of same-sex marriage, had a demonstrable impact on experiences of minority stress and well-being. Grzanka et al. (2020a) conducted interviews with sexual and gender minority individuals living in Tennessee in relation to a law that permits counsellors and therapists in independent practice to deny mental health services to a client based on the practitioner’s “sincerely held principles” (the “conscience clause”). Grzanka and colleagues argue for counselling psychologists to actively engage in opposing “conscience clauses”, which may have a significant impact on the engagement of vulnerable populations with mental health services.

#### 3.1.4. Miscellaneous Populations

Other articles focused on populations such as socioeconomically disadvantaged groups (Articles 41, 63, 71, and 73), including adolescents in resource-constrained settings (Article 45) and people experiencing relative food insecurity (Article 33); employees (Articles 18 and 116), including employees experiencing gender discrimination (Article 24); individuals with physical and/or intellectual disabilities (Articles 40, 64, 68, and 96); refugee populations (Articles 21, 43, and 89); asylum seekers (Article 106); students at school who are disadvantaged (Article 53); people affected by HIV/AIDS (Article 97); marginalised communities (Article 61); historically disadvantaged groups (Article 108); students from culturally and linguistically diverse backgrounds (Article 69); and people who are unemployed (Articles 5 and 8).

For example, Shand et al. (2022) conducted a systematic review to examine whether government policies aimed at addressing unemployment can moderate the effects of unemployment on suicide and self-harm. Their results indicated that unemployment policies can mitigate the relationship between unemployment and suicide, particularly for men. In an analysis of Danish policies in the fields of drug use and treatment, unemployment, and mental health, Bjerge et al. (2020) found that although policies state that multiple factors may impact individuals (such as drug use, unemployment, and mental health), interventions are conceptualised in terms of one type of problem, rather than addressing numerous forces, structures, institutions, and stakeholders.

### 3.2. Age, Gender, and Ethnicity

With regard to age range, seven articles (5.9%) focused on children aged 0–12 years (childhood, including school age), five articles (4.2%) focused on children aged 0–17 years (childhood, school age, and adolescence), three (2.5%) focused on children aged 13–17 years (adolescence), and one (0.8%) focused on individuals aged 13–64 years (adolescence and adulthood including young adulthood, thirties, and middle age). Three articles (2.5%) focused on all ages (0–65+); and 35 articles (29.7%) focused on the age range 18–65+ years only (adulthood, including young adulthood, thirties, middle age, and aged). Notably, 64 (54.2%) articles did not specify an age range.

With respect to gender, 22 articles (74.6%) focused on binary genders (only male and female). Six articles (5.1%) considered transgender people in addition to binary genders (male, transgender, and female). One article (0.8%) focused on female populations only, and one (0.8%) study focused on gender diverse populations. The remaining 88 (74.6%) articles did not specify genders of interest.

The articles focused on a range of ethnic groups, including Latinos (Articles 16 and 76), and Indigenous and Aboriginal populations in Canada (Article 54) and in North America (Article 3). For example, Mitchell and MacLeod (2014) called for the increased commitment of the community mental health profession to the monitoring and advancement of social policy processes that support legitimate community consultation and community wellness in Aboriginal communities. Other articles focused on Asian Americans (Article 113), black and minority ethnic (BME) communities (Article 86), Eastern Asian populations and North American populations (Article 118), Rwandan ethnic groups (Article 48), heterogeneous cultural groups in multicultural societies (Article 75), immigrants and migrants (Articles 12, 70, 85, and 111), and persons belonging to ethnic minorities more broadly (Article 31).

### 3.3. Type of Article and Method

In total, 72 articles (61%) were conceptual papers, including 18 (15.3%) reviews. A total of 46 articles (39%) were empirical articles, including 23 (19.5%) qualitative studies, 18 (15.3%) quantitative studies, and 5 (4.2%) mixed methods studies. The methods described in the empirical articles included interviews and/or focus groups (14, 11.9%), surveys (13, 11%), policy analysis or reviews (7, 5.95%), systematic reviews and/or meta-analysis (4, 3.4%), reviews and analyses of legal judgments (2, 1.7%), and a scoping review (1, 0.8%). The remaining empirical articles used mixed methods. The conceptual articles were varied, including literature reviews (11, 9.3%), conceptual policy analysis/review papers (3, 2.55%), critical reviews (2, 1.7%), narrative reviews (2, 1.7%), conference notes and proceedings (4, 3.4%), comments on articles and commentaries (3, 2.5%), case studies (2, 1.7%), a letter to the editor (1, 0.8%), and a note (1, 0.8%).

### 3.4. Geographical Location

**Figure 2:**
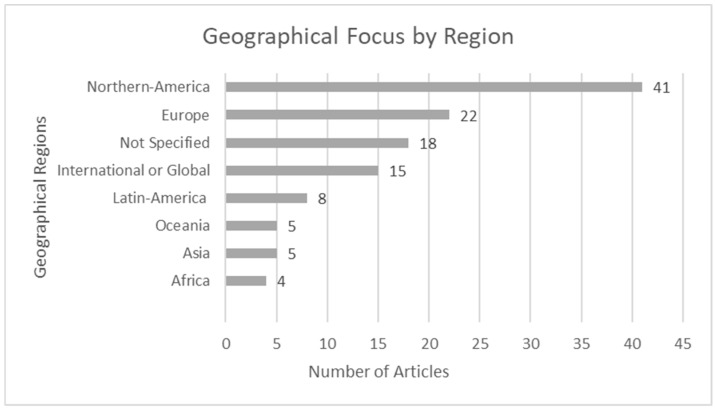
Geographical focus by region/area.

The articles focused on various regions, with some articles spanning multiple jurisdictions. Figure 2 illustrates the distribution of articles by geographical region.

Fifteen articles focused on the global (3) or international level (12), and six articles focused on multiple countries in Europe. Eighteen (15.3%) articles did not specify a geographical focus. In total, 79 (66.9%) articles focused on a single country. The U.S. was a geographical location in focus in 37 (31.4%) articles and the U.K. in 8 (6.8%) articles. The remaining articles focused on Australia (4, 3.4%), Brazil (4, 3.4%), Canada (4, 3.4%), China (3, 2.5%), Portugal (3, 2.5%), Columbia (2, 1.7%), South Africa (2, 1.7%), Argentina (Article 46), Denmark (Article 8), Finland (Article 42), Germany (Article 23), Iran (Article 70), Mexico (Article 82), New Zealand (Article 94), Philippines (Article 76), Rwanda (Article 48), Scotland (Article 80), Switzerland (Article 106), and Uganda (Article 49).

### 3.5. Year of Publication

**Figure 3:**
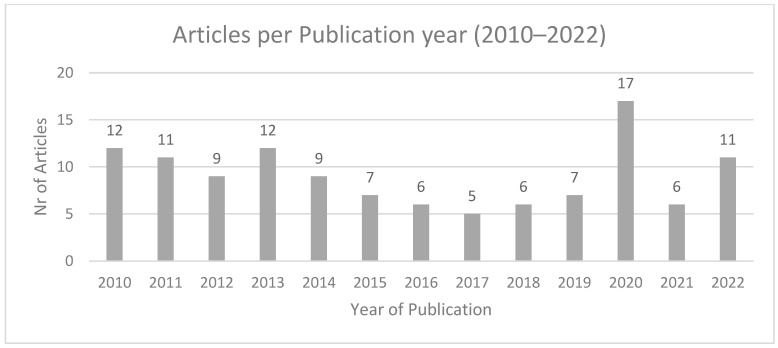
Articles by year of publication.

Figure 3 below presents the publications per year. No trend regarding an increase in publications was identified, notwithstanding the establishment in 2010 of the world’s first governmental institution for the application of behavioural sciences in policy (the UK Behavioural Insights Team). A slight increase in publications is observable for 2020, which potentially may be attributable to the COVID-19 pandemic.

## 4. Discussion

While scoping reviews have been conducted in cognate areas, there is a paucity of reviews of the psychology literature on policy and law. This scoping review addressed this research gap with regard to the extent and nature of psychology’s engagement with public policy and law, particularly in relation to social justice. While the articles identified in this scoping review were diverse with regard to their geographical focus, the research methods employed, and the population in focus, each of the articles engaged with public policy and law, particularly with regard to social justice.

Although the authors of such articles are clearly operating at the macro level and thinking in a ‘macropsychology way’, it is not evident that they conceptualise such work as macropsychology. While the importance of a macropsychology perspective has been discussed elsewhere (see, for example, MacLachlan, 2014, 2017; McVeigh & MacLachlan, 2022), this scoping review is the first to systematically synthesise psychological research at the macro level, adding value to the existing conceptualisation of macropsychology.

A number of articles were directly focused on the integration of psychology with the field of law. For example, Gilfoyle and Dvoskin (2017) provided an overview of APA’s Amicus Curiae Program, which translates psychological research findings to the courts on key public law issues and has therefore advanced the application of psychological research to benefit society. Correspondingly, Liu and Peng (2012) call attention in their article to cultural differences that can impact the use and understanding of laws, emphasising the importance for both psychology and law scholars to examine the ways in which the law’s substantive and procedural standards are applied in different cultural populations.

The scoping review identified 19 articles that were focused on children and/or adolescents. It is crucial for psychologists to adopt a greater focus at the macro level on policies and laws that may impact on the well-being of children and adolescents. For example, a recent study by Weissman et al. (2023) examined if the macrostructural characteristics of the U.S. states, including the cost of living and anti-poverty policies, moderate the associations of low income with brain structure and mental health amongst those in early adolescence. The study used data from the Adolescent Brain and Cognitive Development study from 10,633 9–11-year-old children from 17 U.S. states. The findings indicated that a lower income was associated with a smaller hippocampal volume and higher internalising psychopathology, and these associations were found to be stronger in states that had a higher cost of living. However, in States with a high cost of living that granted higher cash benefits for low-income families, socioeconomic disparities in hippocampal volume were reduced by 34%, so that the association of family income with hippocampal volume was similar to that in the States with the lowest cost of living. The authors asserted that “state-level macrostructural characteristics, including the generosity of anti-poverty policies, are potentially relevant for addressing the relationship of low income with brain development and mental health” (p. 1). A focus at this macro level would allow psychology to have a greater impact with its research findings, to use big data more effectively, and to have more involvement and impact in areas such as organisational and social justice, human rights, equality, and equity (MacLachlan & McVeigh, 2021).

Sixteen articles focused on people with mental health problems and/or substance abuse problems and/or service users. As proposed by WHO Europe (Friedli, 2009, p. v), “A focus on social justice may provide an important corrective to what has been seen as a growing over-emphasis on individual pathology. Mental health is produced socially”. Similarly, the United Nations General Assembly (2017, p. 19) has emphasised that mental health policies need to focus on the “power imbalance” instead of the “chemical imbalance”. As contended by Johnstone et al. (2018) in the *Power Threat Meaning Framework*, although the majority of work on mental health is focused at the individual level, meaning and distress must also be examined at the social, community, and cultural levels, including meaning-based threat responses to the negative operation of power. By recognising power as the basic dynamic underpinning our social relations and our experiences, psychologists can more effectively understand the nature and causes of ‘clinical’ distress and unhappiness and initiate practical ways to alleviate them (Smail, 1995).

From this perspective, mental health is not a medical issue, but rather a social and psychological one, which must be addressed in the context of policies, social justice, equity, and human rights (Kinderman, 2021). Psychologists must therefore turn their attention to policies and laws that impact on the well-being, enjoyment of rights, and social inclusion of people experiencing mental health problems. For example, in the Irish context, while mental health legislation has been enacted, the Irish courts have continued to interpret domestic mental health law in a paternalistic way with regard to the ‘best interests’ of the person, thereby diminishing the autonomy and full personhood of persons with psychosocial disabilities (Department of Health, 2015; Whelan, 2021).

Thirteen articles focused on LGBTQ+ persons. Psychologists must move beyond an individualist perspective to focus on policies and laws that impact the well-being, rights, and inclusion of LGBTQ+ people. For example, a recent national study on the mental health and well-being of the LGBTQI+ communities in Ireland (Higgins et al., 2024, p. 60) found that LGBTQI+ people took strength from positive societal developments, particularly recent progressive policies, which were viewed as ‘coping ability boosters’. Indeed, both national and local anti-bullying and anti-discrimination policies that explicitly refer to sexuality and gender have been found to increase feelings of safety amongst sexual and gender minority youth and lower their adverse experiences at school (Költő et al., 2021). Similarly, Lampe et al. (2024) have called for more research on upstream social and policy factors that impact the health and lack of social support experienced by LGBTQ+ persons.

A small number of articles focused on other structurally vulnerable populations such as persons with physical and/or intellectual disabilities, people affected by HIV/AIDS, refugee populations, and asylum seekers. Psychologists must move beyond the individual level to address macro level factors that influence the well-being of vulnerable groups. Mental health problems are more prevalent amongst structurally vulnerable groups, including those living in poverty and people with HIV/AIDS, whereby mental health outcomes progressively worsen with lower social position due to the social gradient of mental health (Mannan et al., 2013; Mezzina et al., 2022). It is well established that the social determinants of health—‘the causes of the causes’—significantly influence health and are determined by social policies (Braveman & Gottlieb, 2014), and it is to these macro level determinants that psychology must therefore devote greater attention.

### Limitations

The very broad scope of this review was challenging, and the search was therefore limited to the APA PsycInfo database and to the time scale of 2010 onwards. The findings of this review should therefore be interpreted with regard to these caveats in addition to those outlined below.

Although this study used systematic scoping review methods, it is important to note that the findings of this study are derived from a scoping review rather than a systematic review or meta-analysis. However, scoping reviews are appropriate for research areas that have not been extensively reviewed, whereby such reviews can be used as a preliminary assessment of the extent, scope, and nature of literature in a particular area, alongside gaps in the literature (Premachandra & Lewis, 2022).

The wide scope of the research area and the need to refine the search strategy resulted in the exclusion of articles in cognate areas such as psychiatry publications (see exclusion criteria presented in Table 1), therefore limiting the inclusion of articles focused on particular vulnerable populations such as people subject to involuntary detention. Many articles in the area of forensic psychology were also excluded, such as those focused on the rights of incarcerated persons. Furthermore, with regard to papers returned from multidisciplinary journals, articles were only included if the first author was working in the field of psychology, as determined by the authors’ affiliations.

It is also important to consider that this review included only publications with a particular focus on social justice. Publications addressing psychology at the macro level were therefore excluded where this focus was not evident. However, a focus on social justice was adopted due to the importance of law and policies that are rights-based and well rooted in the principles of fairness and social inclusion. Social justice influences the way people live and their resulting risk of morbidity and premature death and is therefore literally “a matter of life and death” (WHO & WHO Commission on Social Determinants of Health, 2008, p. i).

## 5. Conclusions

Macro-level domains such as public policy and public health have traditionally not been a focus of the field of psychology (Rami et al., 2022). It is crucial for psychology to adopt a greater focus on macro-level factors at the population level, including policies and laws, that impact on well-being. Similarly, at the international level, by translating our knowledge, skills, methods and models into practical proposals, psychologists can significantly contribute to global policy content and processes (Bullock et al., 2023). This is illustrated by psychologists’ contribution to promoting social justice through policy, for example, by developing instruments to evaluate the extent to which marginalised groups are addressed in policy content (EquiFrame) or involved in policy processes (EquIPP) (MacLachlan et al., 2012; Mannan et al., 2012; McVeigh et al., 2024). In addition to the need for psychology’s greater involvement in policy content and processes, psychology can make valuable contributions to law. As proposed by Sales and Krauss (2015), while psychologists have been making significant contributions to the understanding of law throughout the past number of decades, we need to take a step back and reconceptualise how we can enhance psychology’s contribution to the field of law.

It is imperative for psychology’s involvement at the macro level to be guided by the principle of social justice. Psychology must zoom out from the individual level to also examine the systems, institutions, and policies that perpetuate marginalisation and unfairness; otherwise, it risks focusing on the symptoms of social injustice without addressing the root causes (MacLachlan, 2017). Psychology as a field can contribute greatly to promoting social justice, requiring psychologists to systematically apply their knowledge to social issues (Vasquez, 2012). Indeed, “justice requires psychological-mindedness” (Melton, 1995, p. xxii).

A macropsychology perspective supplements micro- and meso-perspectives in psychology, connecting these in a meta-system (MacLachlan et al., 2019). Psychology therefore needs to both ‘zoom in’ and ‘zoom out’ to develop more balanced, complete, and meaningful understandings of psychological phenomena (Van de Vliert et al., 2023). As asserted by Tropp (2023), “Collectively, as a discipline, we have only begun to cultivate the presence and influence we could potentially have in the public sphere, speaking to the broader relevance of psychological research for people’s lives and society at large”. To foster this influence in the public sphere and to develop a more meaningful understanding of psychological well-being, psychology will need to focus on macro-level factors that affect our well-being and how psychology can, in turn, be wielded to influence them.

## Figures and Tables

**Table 1:** Inclusion and exclusion criteria.

**Inclusion Criteria:**	
Publication Year:	From 2010 to present.
Language:	The search was conducted in English. Articles not in English were translated.
Types of Research:	Psychology publications, including quantitative, qualitative, mixed methods, and theoretical papers.
Types of Documents:	Peer-reviewed journal articles, reports, reviews, and book chapters, published in a psychology journal or a multidisciplinary journal where the first author is working in the field of psychology.
Research Focus:	– Publications focused on psychology AND macro-level factors, including structures, systems, institutions, policies, and laws, at the national, regional, intergovernmental, and supranational levels, with a focus on social justice. – Behavioural insights/behavioural governance, with a focus on social justice.
**Exclusion Criteria:**	
Publication Year:	Prior to 2010.
Types of Research:	– Protocols. – Testing measures.
Types of Documents:	– Book reviews. – Pre-prints. – Abstracts. – Bibliographies. – Editorials. – Letters to editors. – A corrigendum that does not revise the actual content of articles. – Award addresses. – Articles not published in a peer-reviewed psychology journal or a multidisciplinary journal where the first author is working in the field of psychology.
Research Focus:	– Psychology AND macro-level factors without a focus on social justice. – Behavioural insights/behavioural governance, without a focus on social justice. – Psychiatry publications.

**Table 2:** Search terms for scoping review.

Main Search Terms: 1 AND 2 Minor Search Terms: 3
((Psychology OR Psychological OR Psychologist OR Psychologists OR “Mental Health”) (Macro OR Population OR Populations OR “Sustainable Development Goal” OR “Sustainable Development Goals” OR SDGs OR Government OR Governments OR Country OR Countries OR Region* OR International OR Intergovernmental OR Supernational OR Supranational OR Global* OR “Social Structure” OR “Social Structures” OR System OR Systems OR Institution* OR Policy OR Policies OR Law OR Laws OR Legal* OR Litigation OR Judicial OR Judiciary OR Legis* OR Statut*))
3. (“Behavioral insights” OR “Behavioural insights” OR “Behavioral governance” OR “Behavioural governance” OR “Behavioral government” OR “Behavioural government”)

**Table 3:** Focus of articles—populations.

Populations	Subpopulations	Number of Articles	Article Number (Article Numbers Correspond with those in Supplementary File S2)
*Children and/or adolescents*		19, 16.1%	2, 7, 11, 26, 37, 44, 46, 55, 57, 65, 78, 80, 84, 91, 94, 100, 114, 115, 117
	Children	4	26, 78, 80, 94
	Children and adolescents	4	2, 7, 46, 57
	Adolescents	1	114
	Left-behind children from rural primary schools in remote areas of western China	1	11
	Children with special healthcare needs	1	37
	Youth with obesity	1	115
	Children and/or adolescents and their families	2	65, 91
	Families experiencing separation	1	117
	Families with both parental mental health issues and child protection concerns	1	84
	Professional practitioners such as psychologists working with children	1	100
	Legal professionals working with lawsuits involving children	1	44
	Players in the judicial sector involving children	1	55
*Ethnic minorities*		15, 12.71%	3, 12, 16, 31, 48, 54, 69, 70, 75, 76, 85, 86, 111, 113, 118
	Latino populations	2	16, 76
	Indigenous and Aboriginal (North Americas and Canada)	2	3, 54
	Asian Americans	1	113
	Black and minority ethnic (BME) communities	1	86
	Eastern Asian and North American populations	1	118
	Rwandan ethnic groups	1	48
	Heterogeneous cultural groups in multicultural societies	1	75
	Immigrants and migrants	4	12, 70, 85, 111
	Ethnic minorities more broadly	2	31, 69
*People with mental health problems and/or substance abuse problems and/or mental health service users*		15, 12.71%	1, 6, 8, 10, 19, 22, 23, 27, 36, 47, 51, 77, 79, 88, 90
	People with mental health problems and/or substance abuse problems and/or treatment service users	8	8, 19, 22, 23, 27,77, 88, 90
	Individuals found not criminally responsible on account of mental disorder	1	79
	Mental health service users and people subject to (involuntary) detention	2	1, 10
	Mental health system stakeholders, service users and advocates more broadly	4	6, 36, 47, 51
	People with mental, neurological and substance use disorders in low- and middle-income countries	1	27
*LGBTQ+ persons*		13, 11.01%	4, 9, 13, 15, 17, 20, 32, 34, 62, 87, 99, 103, 104
	Individuals in same-sex relationships	1	4
	Lesbian, Gay, and Bisexual (LGB) individuals	2	32, 104
	LGBTQ individuals and communities	2	13, 62
	The Lesbian, Gay, Bisexual, Transgender, and Queer (LGBTQ+) community	1	87
	LGBTQ+ clients and professional practitioners such as psychologists	2	34, 103
	Sexual minority (LGBQ+) college students	1	20
	Sexual and gender minority individuals living in Tennessee	1	9
	Transgender and gender-diverse (TGD) individuals	2	17, 99
	Parents of trans and gender-diverse (TGD) youth	1	15
*Miscellaneous populations*		27, 22.88%	5, 14, 18, 21, 24, 28, 33, 40, 41, 43, 45, 58, 61, 63, 64, 66, 68, 69, 71, 73, 89, 96, 97, 106, 107, 108, 116
	Asylum seekers	1	106
	Refugee populations	3	21, 43, 89
	Individuals with physical or intellectual disabilities	3	40, 68, 96
	Employees	2	18, 116
	Employees experiencing gender discrimination	1	24
	People who are unemployed	1	5
	Disenfranchised individuals living with mental health disorder and/or physical disabilities	1	64
	Citizens affected by HIV/AIDS	1	97
	Populations with lived experience of the Movement for Global Mental Health in low- and middle-income countries	1	107
	Globally socioeconomically disadvantaged	1	71
	Residents in socioeconomically disadvantaged neighbourhoods	1	41
	Marginalised communities	1	61
	Socioeconomically disadvantaged individuals	2	63, 73
	Adolescents in resource-constrained settings	1	45
	School students who are disadvantaged	1	58
	Adults living in a rural and urban community	1	28
	People experiencing relative food insecurity	1	33
	Historically disadvantaged groups	1	108
	Students from culturally and linguistically diverse backgrounds	1	69
	Ageing populations	1	14
	Women who are subject to detention	1	66
*Other*	Professional practitioners	15, 12.71%	25, 30, 38, 39, 52, 56, 60, 67, 72, 83, 92, 95, 98, 101, 105
	Public policies/laws/rights	14, 11.86%	29, 35, 42, 49, 50, 59, 74, 81, 82, 93, 102, 109, 110, 112

## Data Availability

To obtain further details on the analysis reported in this study, please contact the authors.

## Supplementary Materials

The following supporting information can be downloaded at https://www.mdpi.com/article/10.3390/bs15030350/s1: Supplementary File S1: PRISMA-ScR checklist; Supplementary File S2: Data extraction sheet; Supplementary File S3: Database electronic search strategy. References (Abreu et al., 2021, 2022; Agrusti et al., 2020; Alfaro & Martin, 2015; Aranda, 2016; Baker & Brassard, 2019; Barrera Rojas & Baeza Ruiz, 2021; Battams & Henderson, 2012; Bendat, 2014; Benelli, 2016; Borg, 2010; Brodsky et al., 2013; Browne et al., 2020; Campbell & Steel, 2015; Carew et al., 2010; Chaplin & Taggart, 2012; Chen, 2020; Chhabra & Kapadia, 2023; Christie & Montiel, 2013; Cook & Roesch, 2012; Cosgrove et al., 2020b; Cowan et al., 2011; Crocker et al., 2010; Das, 2019; Davies et al., 2010; De Fátima Guareschi et al., 2010; C. De Freitas et al., 2014; D. F. De Freitas et al., 2018; De la Peña et al., 2019; DeBoer et al., 2022; Dopp & Lantz, 2020; Elgar et al., 2021; Evans & Russell-Mayhew, 2020; Fine, 2013; C. B. Fisher, 2013; J. R. W. Fisher & de Mello, 2011; Forsman et al., 2015; Fullen et al., 2019; Funk et al., 2012; Furlan & Pelissari, 2013; García-Vázquez et al., 2020; Gerlinger et al., 2019; Girvan & Marek, 2016; Gómez, 2010; Grey et al., 2013; Grzanka et al., 2020b; Hall & Yee, 2012; Hatzenbuehler, 2010; Hatzenbuehler et al., 2017; Hennes & Dang, 2021; Javakhishvili et al., 2020; Johnson, 2010; Kaczkowski et al., 2022; Kamody et al., 2022; Kenny et al., 2017; Kinscherff & Grisso, 2013; Kleintjes et al., 2012; Kourgiantakis et al., 2022; Lange & Williams, 2011; Lea, 2021; Lenta & Zaldúa, 2020; Leslie & Manchester, 2011; Lessard & Lawrence, 2022; Li & Hui, 2020; Major, 2018; Melton, 2010; Migacheva, 2015; Moreno et al., 2020; Moss & Vollhardt, 2016; Mowat, 2020; Muniz Neto et al., 2014; Newbigging & Ridley, 2018; Newbigging et al., 2015; O’Donnell, 2012; Oster et al., 2016; Österman et al., 2014; Perucchi et al., 2011; Petersen et al., 2016; Pettigrew, 2011; Pinheiro & Sousa, 2020; Riggle et al., 2010; Salas et al., 2013; Sanchez-Mazas, 2015; Scanlon & Adlam, 2013; Spears, 2010; Steel et al., 2011/2015; Toquero, 2021; Torres et al., 2018; Triana et al., 2019; Tricco et al., 2018; Uribe Aramburo, 2011; Valentim, 2013; Vodanovich & Piotrowski, 2011; Ward et al., 2018; Wolf et al., 2013; Woodford et al., 2018; Ximenes et al., 2015; Yadegarfard & Bahramabadian, 2014; Zayas & Bradlee, 2014) are cited in the Supplementary Materials.

## References

[B1-behavsci-15-00350] Abreu R. L., Sostre J. P., Gonzalez K. A., Lockett G. M., Matsuno E. (2021). ‘I am afraid for those kids who might find death preferable’: Parental figures’ reactions and coping strategies to bans on gender affirming care for transgender and gender diverse youth. Psychology of Sexual Orientation and Gender Diversity.

[B2-behavsci-15-00350] Abreu R. L., Sostre J. P., Gonzalez K. A., Lockett G. M., Matsuno E., Mosley D. V. (2022). Impact of gender-affirming care bans on transgender and gender diverse youth: Parental figures’ perspective. Journal of Family Psychology.

[B3-behavsci-15-00350] Agrusti T., Bohn J., Dunn E., Bell C., Ziegler A. (2020). The story so far: A mixed-methods evaluation of county-level behavioral health needs, policies, and programs. Social Work in Mental Health.

[B4-behavsci-15-00350] Alfaro J., Martin M. P. (2015). Proceso y oportunidades de la transferencia del conocimiento desde la psicología comunitaria a las políticas públicas = Process and opportunities for knowledge transfer from community psychology to public policy. Universitas Psychologica.

[B5-behavsci-15-00350] Andrawis A., Tapa J., Vlaev I., Read D., Schmidtke K. A., Chow E. P. F., Lee D., Fairley C. K., Ong J. J. (2022). Applying behavioural insights to HIV prevention and management: A scoping review. Current HIV/AIDS Reports.

[B6-behavsci-15-00350] Aranda R. (2016). Living in the shadows: Plight of the undocumented. Journal of Clinical Psychology.

[B7-behavsci-15-00350] Arksey H., O’Malley L. (2005). Scoping studies: Towards a methodological framework. International Journal of Social Research Methodology.

[B8-behavsci-15-00350] Baker A. J. L., Brassard M. (2019). Predictors of variation in sate reported rates of psychological maltreatment: A survey of statutes and a call for change. Child Abuse & Neglect.

[B9-behavsci-15-00350] Barrera Rojas M. A., Baeza Ruiz A. (2021). La salud mental como derecho humano en Quintana Roo, México Análisis desde la disciplina de la política pública = Mental health as a human right in Quintana Roo, Mexico Analysis from the discipline of public policy. Interdisciplinaria Revista de Psicología y Ciencias Afines.

[B10-behavsci-15-00350] Battams S., Henderson J. (2012). The right to health, international human rights legislation and mental health policy and care practices for people with psychiatric disability. Psychiatry, Psychology and Law.

[B11-behavsci-15-00350] Bendat M. (2014). In name only? Mental health parity or illusory reform. Psychodynamic Psychiatry.

[B12-behavsci-15-00350] Benelli S. J. (2016). Risco e vulnerabilidade como analisadores nas políticas públicas sociais: Uma análise crítica = Risk and vulnerability as criteria to evaluate social and public policies: A critical analysis. Estudos de Psicologia.

[B13-behavsci-15-00350] Bielsa V. C., Braddick F., Jané-Llopis E., Jenkins R., Puras D. (2010). Child and adolescent mental health policies, programmes and infrastructures across Europe. International Journal of Mental Health Promotion.

[B14-behavsci-15-00350] Bjerge B., Christensen L., Oute J. (2020). Complex cases—Complex representations of problems. International Journal of Drug Policy.

[B15-behavsci-15-00350] Borg M. B. J. (2010). Disability, social policy and the burden of disease: Creating an ‘assertive’ community mental health system in New York. Psychology.

[B16-behavsci-15-00350] Braveman P., Gottlieb L. (2014). The social determinants of health: It’s time to consider the causes of the causes. Public Health Reports.

[B17-behavsci-15-00350] Brodsky S. L., Neal T. M. S., Jones M. A. (2013). A reasoned argument against banning psychologists’ involvement in death penalty cases. Ethics & Behavior.

[B18-behavsci-15-00350] Browne N., Zlotowitz S., Alcock K., Barker C. (2020). Practice to policy: Clinical psychologists’ experiences of macrolevel work. Professional Psychology: Research and Practice.

[B19-behavsci-15-00350] Bullock M., Hofgaard T. L., Benito E., Flattau P., Clinton A., Shealy C., Kapadia S., Shealy C., Bullock M., Kapadia S. (2023). Policy: Why and how to become engaged as an international policy psychologist. Going global: How psychologists can meet a world of need.

[B20-behavsci-15-00350] Callaghan J. E. M., Fellin L. C., Warner-Gale F. (2017). A critical analysis of child and adolescent mental health services policy in England. Clinical Child Psychology and Psychiatry.

[B21-behavsci-15-00350] Campbell E. J., Steel E. J. (2015). Mental distress and human rights of asylum seekers. Journal of Public Mental Health.

[B22-behavsci-15-00350] Carew D., Birkin R., Booth D. (2010). Employment, policy and social inclusion. The Psychologist.

[B23-behavsci-15-00350] Carr S. C., MacLachlan M. (2014). Humanitarian work psychology. The Psychologist.

[B24-behavsci-15-00350] Chaplin E., Taggart L. (2012). England and Northern Ireland policy and law update relating to mental health and intellectual disability. Advances in Mental Health and Intellectual Disabilities.

[B25-behavsci-15-00350] Chen H. (2020). Improvement of mental health of labourers through the publicity of social security law. Revista Argentina de Clínica Psicológica.

[B26-behavsci-15-00350] Chhabra B., Kapadia S. (2023). Mental health policies in queer community: Are we doing enough?. Journal of Loss and Trauma.

[B27-behavsci-15-00350] Christie D. J., Montiel C. J. (2013). Contributions of psychology to war and peace. American Psychologist.

[B28-behavsci-15-00350] Cook A. N., Roesch R. (2012). ‘Tough on crime’ reforms: What psychology has to say about the recent and proposed justice policy in Canada. Canadian Psychology/Psychologie Canadienne.

[B29-behavsci-15-00350] Cosgrove L., Mills C., Karter J. M., Mehta A., Kalathil J. (2020a). A critical review of the Lancet Commission on global mental health and sustainable development: Time for a paradigm change. Critical Public Health.

[B30-behavsci-15-00350] Cosgrove L., Morrill Z., Karter J. M., Valdes E., Cheng C.-P. (2020b). The cultural politics of mental illness: Toward a rights-based approach to global mental health. Community Mental Health Journal.

[B31-behavsci-15-00350] Cowan S., Banks D., Crawshaw P., Clifton A. (2011). Mental health service user involvement in policy development: Social inclusion or disempowerment?. Mental Health Review Journal.

[B32-behavsci-15-00350] Crocker A. G., Nicholls T. L., Côté G., Latimer E. A., Seto M. C. (2010). Individuals found not criminally responsible on account of mental disorder: Are we providing equal protection and equivalent access to mental health services across Canada?. Canadian Journal of Community Mental Health.

[B33-behavsci-15-00350] Cronin T., Gouda P., McDonald C., Hallahan B. (2017). A comparison of mental health legislation in five developed countries: A narrative review. Irish Journal of Psychological Medicine.

[B34-behavsci-15-00350] Das B. (2019). Mental health trauma treatment within the current Mediterranean refugee crisis. International Journal for the Advancement of Counselling.

[B35-behavsci-15-00350] Davies E., Crothers C., Hanna K. (2010). Preventing child poverty: Barriers and solutions. New Zealand Journal of Psychology.

[B40-behavsci-15-00350] DeBoer J. L., Allouche S. F., Vasquez J. I., Rhodes J. (2022). Equitable practices in school mental health. Psychology in the Schools.

[B36-behavsci-15-00350] De Fátima Guareschi N. M., de Lara L., Adegas M. A. (2010). Políticas públicas entre o sujeito de direitos e o homo œconomicus = Social psychology and public policies: Between the subject’s rights and the homo œconomicus. Psico.

[B37-behavsci-15-00350] De Freitas C., García-Ramirez M., Aambø A., Buttigieg S. C. (2014). Transforming health policies through migrant user involvement: Lessons learnt from three European countries. Psychosocial Intervention.

[B38-behavsci-15-00350] De Freitas D. F., Fern Jesus M., Ferreira P. D., Coimbra S., Teixeira P. M., de Moura A., Gato J., Marques S. C., Fontaine A. M. (2018). Psychological correlates of perceived ethnic discrimination in Europe: A meta-analysis. Psychology of Violence.

[B39-behavsci-15-00350] De la Peña C. M., Pineda L., Punsky B. (2019). Working with parents and children separated at the border: Examining the impact of the zero tolerance policy and beyond. Journal of Child & Adolescent Trauma.

[B41-behavsci-15-00350] Department of Health (Ireland) (2015). Report of the expert group review of the Mental Health Act, 2001.

[B42-behavsci-15-00350] Dopp A. R., Lantz P. M. (2020). Moving upstream to improve children’s mental health through community and policy change. Administration and Policy in Mental Health and Mental Health Services Research.

[B43-behavsci-15-00350] Elgar F. J., Pickett W., Pförtner T.-K., Gariépy G., Gordon D., Georgiades K., Davison C., Hammami N., MacNeil A. H., Da Silva M. A., Melgar-Quiñonez H. R. (2021). Relative food insecurity, mental health and wellbeing in 160 countries. Social Science & Medicine.

[B44-behavsci-15-00350] Evans H., Russell-Mayhew S. (2020). The responsibility of Canadian counselling psychology to reach systems, organizations, and policy-makers. Canadian Journal of Counselling and Psychotherapy.

[B45-behavsci-15-00350] Fine M. (2013). Echoes of Bedford: A 20-year social psychology memoir on participatory action research hatched behind bars. American Psychologist.

[B46-behavsci-15-00350] Fisher C. B. (2013). Human rights and psychologists’ involvement in assessments related to death penalty cases. Ethics & Behavior.

[B47-behavsci-15-00350] Fisher J. R. W., de Mello M. C. (2011). Using the World Health Organization’s 4S-Framework to strengthen national strategies, policies and services to address mental health problems in adolescents in resource-constrained settings. International Journal of Mental Health Systems.

[B48-behavsci-15-00350] Focarelli C. (2012). International law as social construct: The struggle for global justice.

[B49-behavsci-15-00350] Forberger S., Reisch L., Kampfmann T., Zeeb H. (2019). Nudging to move: A scoping review of the use of choice architecture interventions to promote physical activity in the general population. International Journal of Behavioral Nutrition and Physical Activity.

[B50-behavsci-15-00350] Forsman A. K., Fredén L., Lindqvist R., Wahlbeck K. (2015). Contribution of the Nordic School of Public Health to the public mental health research field: A selection of research initiatives, 2007–2014. Scandinavian Journal of Public Health.

[B51-behavsci-15-00350] Friedli L. (2009). Mental health, resilience and inequalities.

[B52-behavsci-15-00350] Fullen M. C., Wiley J. D., Morgan A. A. (2019). The Medicare mental health coverage gap: How licensed professional counselors navigate Medicare-ineligible provider status. The Professional Counselor.

[B53-behavsci-15-00350] Funk M., Drew N., Knapp M. (2012). Mental health, poverty and development. Journal of Public Mental Health.

[B54-behavsci-15-00350] Furlan V., Pelissari M. A. (2013). Psicologia e os contextos socio-político-cultural e das políticas sociais no século XXI = Psychology and socio-political-cultural and social policies contexts in the XXI century. Psicologia: Ciência e Profissâo.

[B55-behavsci-15-00350] García-Vázquez E., Reddy L., Arora P., Crepeau-Hobson F., Fenning P., Hatt C., Hughes T., Jimerson S., Malone C., Minke K., Radliff K., Raines T., Song S., Strobach K. V. (2020). School psychology unified antiracism statement and call to action. School Psychology Review.

[B56-behavsci-15-00350] Gerlinger G., Deister A., Heinz A., Koller M., Müller S., Steinert T., Pollmächer T. (2019). Nach der Reform ist vor der Reform: Ergebnisse der Novellierungsprozesse der Psychisch-Kranken-Hilfe-Gesetze der Bundesländer = After the reform is before the reform: Results of the amendment processes of mental health law in German federal states. Der Nervenarzt.

[B57-behavsci-15-00350] Gilfoyle N., Dvoskin J. A. (2017). APA’s amicus curiae program: Bringing psychological research to judicial decisions. American Psychologist.

[B58-behavsci-15-00350] Girvan E., Marek H. J. (2016). Psychological and structural bias in civil jury awards. Journal of Aggression, Conflict and Peace Research.

[B59-behavsci-15-00350] Gómez S. A. (2010). La salud mental a la luz de la Constitución colombiana: Análisis de algunas sentencias de la Corte Constitucional, 1992–2009 = Mental health in the light of the Colombian Constitution: Analysis of some of the sentences of the Constitutional Court, 1992–2009. Revista Colombiana de Psiquiatría.

[B60-behavsci-15-00350] Grey T., Sewell H., Shapiro G., Ashraf F. (2013). Mental health inequalities facing UK minority ethnic populations: Causal factors and solutions. Journal of Psychological Issues in Organizational Culture.

[B61-behavsci-15-00350] Grzanka P. R., DeVore E. N., Frantell K. A., Miles J. R., Spengler E. S. (2020a). Conscience clauses and sexual and gender minority mental health care: A case study. Journal of Counseling Psychology.

[B62-behavsci-15-00350] Grzanka P. R., Spengler E. S., Miles J. R., Frantell K. A., DeVore E. N. (2020b). ‘Sincerely held principles’ or prejudice? The Tennessee Counseling Discrimination Law. The Counseling Psychologist.

[B63-behavsci-15-00350] Hall G. C. N., Yee A. H. (2012). US mental health policy: Addressing the neglect of Asian Americans. Asian American Journal of Psychology.

[B64-behavsci-15-00350] Hallsworth M., Egan M., Rutter J., McCrae J. (2018). Behavioural government: Using behavioural science to improve how governments make decisions.

[B65-behavsci-15-00350] Hatzenbuehler M. L. (2010). Social factors as determinants of mental health disparities in LGB populations: Implications for public policy. Social Issues and Policy Review.

[B66-behavsci-15-00350] Hatzenbuehler M. L., Prins S. J., Flake M., Philbin M., Frazer M. S., Hagen D., Hirsch J. (2017). Immigration policies and mental health morbidity among Latinos: A state-level analysis. Social Science & Medicine.

[B67-behavsci-15-00350] Hennes E. P., Dang L. (2021). The devil we know: Legal precedent and the preservation of injustice. Policy Insights from the Behavioral and Brain Sciences.

[B68-behavsci-15-00350] Higgins A., Downes C., O’Sullivan K., de Vries J., Molloy R., Monahan M., Keogh B., Doyle L., Begley T., Corcoran P. (2024). Being LGBTQI+ in Ireland; The national study on the mental health and wellbeing of the LGBTQI+ communities in Ireland.

[B69-behavsci-15-00350] Javakhishvili J. D., Ardino V., Bragesjö M., Kazlauskas E., Olff M., Schäfer I. (2020). Trauma-informed responses in addressing public mental health consequences of the COVID-19 pandemic: Position paper of the European Society for Traumatic Stress Studies (ESTSS). European Journal of Psychotraumatology.

[B70-behavsci-15-00350] Johnson J. (2010). Recognition of the nonhuman: The psychological minefield of transgender inequality in the law. Law & Psychology Review.

[B71-behavsci-15-00350] Johnstone L., Boyle M., Cromby J., Dillon J., Harper D., Kinderman P., Longden E., Pilgrim D., Read J. (2018). The power threat meaning framework: Towards the identification of patterns in emotional distress, unusual experiences and troubled or troubling behaviour, as an alternative to functional psychiatric diagnosis.

[B72-behavsci-15-00350] Jones R., Whitehead M. (2018). ‘Politics done like science’: Critical perspectives on psychological governance and the experimental state. Environment and Planning D: Society and Space.

[B73-behavsci-15-00350] Kaczkowski W., Li J., Cooper A. C., Robin L. (2022). Examining the relationship between LGBTQ-supportive school health policies and practices and psychosocial health outcomes of lesbian, gay, bisexual, and heterosexual students. LGBT Health.

[B74-behavsci-15-00350] Kamody R. C., Kamody E. S., Rosenthal A., Olezeski C. L. (2022). Optimizing medical-legal partnerships in pediatric psychology to reduce health disparities. Journal of Pediatric Psychology.

[B75-behavsci-15-00350] Kenny M. C., Abreu R. L., Marchena M. T., Helpingstine C., Lopez-Griman A., Mathews B. (2017). Legal and clinical guidelines for making a child maltreatment report. Professional Psychology: Research and Practice.

[B76-behavsci-15-00350] Kinderman P., MacLachlan M., McVeigh J. (2021). From chemical imbalance to power imbalance: A macropsychology perspective on mental health. Macropsychology: A population science for sustainable development goals.

[B77-behavsci-15-00350] Kinscherff R. T., Grisso T. J. (2013). Human rights violations and Standard 102: Intersections with human rights law and applications in juvenile capital cases. Ethics & Behavior.

[B78-behavsci-15-00350] Kitafuna K. B. (2022). A critical overview of mental health-related beliefs, services and systems in Uganda and recent activist and legal challenges. Community Mental Health Journal.

[B79-behavsci-15-00350] Kleintjes S., Lund C., Swartz L. (2012). South African mental health care service user views on priorities for supporting recovery: Implications for policy and service development. Disability and Rehabilitation: An International, Multidisciplinary Journal.

[B80-behavsci-15-00350] Knapp S. J., Fingerhut R., Knapp S. J., Fingerhut R. (2024). Social justice. Practical ethics for psychologists: A positive approach.

[B81-behavsci-15-00350] Kourgiantakis T., McNeil S. R., Hussain A., Logan J., Ashcroft R., Lee E., Williams C. C. (2022). Social work’s approach to recovery in mental health and addiction policies: A scoping review. Social Work in Mental Health.

[B82-behavsci-15-00350] Költő A., Vaughan E., O’Sullivan L., Kelly C., Saewyc E. M., Nic Gabhainn S. (2021). LGBTI+ Youth in Ireland and across Europe: A two-phased landscape and research gap analysis.

[B83-behavsci-15-00350] Lampe N. M., Barbee H., Tran N. M., Bastow S., McKay T. (2024). Health disparities among Lesbian, Gay, Bisexual, Transgender, and Queer older adults: A structural competency approach. The International Journal of Aging and Human Development.

[B84-behavsci-15-00350] Lange R., Williams A. S. (2011). Linking adults’ problems with children’s pain: Legal, ethical and clinical issues. Psychiatry, Psychology and Law.

[B85-behavsci-15-00350] Lea S. E. G. (2021). Debt and overindebtedness: Psychological evidence and its policy implications. Social Issues and Policy Review.

[B86-behavsci-15-00350] Lenta M. M., Zaldúa G. (2020). Vulnerabilidad y Exigibilidad de Derechos: La Perspectiva de Niños, Niñas y Adolescentes = Vulnerability and rights enforceability: The perspective of children and adolescents. Psykhe: Revista de la Escuela de Psicología.

[B87-behavsci-15-00350] Leslie L. M., Manchester C. F. (2011). Work–family conflict is a social issue not a women’s issue. Industrial and Organizational Psychology: Perspectives on Science and Practice.

[B88-behavsci-15-00350] Lessard L. M., Lawrence S. E. (2022). Weight-based disparities in youth mental health: Scope, social underpinnings, and policy implications. Policy Insights from the Behavioral and Brain Sciences.

[B89-behavsci-15-00350] Li X., Hui X. (2020). Effect of government policies on mental health of left-behind children. Revista Argentina de Clínica Psicológica.

[B90-behavsci-15-00350] Liu B.-H., Peng K.-P. (2012). Challenge and contribution of cultural psychology to empirical legal studies. Acta Psychologica Sinica.

[B91-behavsci-15-00350] Lund C., Kleintjes S., Cooper S., Petersen I., Bhana A., Flisher A. J. (2011). Challenges facing South Africa’s mental health care system: Stakeholders’ perceptions of causes and potential solutions. International Journal of Culture and Mental Health.

[B92-behavsci-15-00350] MacLachlan M. (2014). Macropsychology, policy, and global health. American Psychologist.

[B93-behavsci-15-00350] MacLachlan M. (2017). Still too POSH to push for structural change? The need for a macropsychology perspective. Industrial and Organizational Psychology: Perspectives on Science and Practice.

[B94-behavsci-15-00350] MacLachlan M., Amin M., Mannan H., El Tayeb S., Bedri N., Swartz L., Munthali A., Van Rooy G., McVeigh J. (2012). Inclusion and human rights in health policies: Comparative and benchmarking analysis of 51 policies from Malawi, Sudan, South Africa and Namibia. PLoS ONE.

[B95-behavsci-15-00350] MacLachlan M., McVeigh J., MacLachlan M., McVeigh J. (2021). Macropsychology: Definition, scope and conceptualisation. Macropsychology: A population science for Sustainable Development Goals.

[B96-behavsci-15-00350] MacLachlan M., McVeigh J., Huss T., Mannan H., O’Doherty K. C., Hodgetts D. (2019). Macropsychology: Challenging and changing social structures and systems to promote social inclusion. The sage handbook of applied social psychology.

[B97-behavsci-15-00350] Magor-Blatch L. (2011). Beyond zero tolerance: Providing a framework to promote social justice and healthy adolescent development. The Australian Educational and Developmental Psychologist.

[B98-behavsci-15-00350] Major L. H. (2018). Mental health news: How frames influence support for policy and civic engagement intentions. Journal of Health Communication.

[B99-behavsci-15-00350] Mannan H., ElTayeb S., MacLachlan M., Amin M., McVeigh J., Munthali A., Van Rooy G. (2013). Core concepts of human rights and inclusion of vulnerable groups in the mental health policies of Malawi, Namibia, and Sudan. International Journal of Mental Health Systems.

[B100-behavsci-15-00350] Mannan H., McVeigh J., Amin M., MacLachlan M., Swartz L., Munthali A., VanRooy G. (2012). Core concepts of human rights and inclusion of vulnerable groups in the disability and rehabilitation policies of Malawi, Namibia, Sudan, and South Africa. Journal of Disability Policy Studies.

[B101-behavsci-15-00350] Marmot M. (2017). Social justice, epidemiology and health inequalities. European Journal of Epidemiology.

[B102-behavsci-15-00350] McKnight K. M., Sechrest L., McKnight P. E. (2005). Psychology, psychologists, and public policy. Annual Review of Clinical Psychology.

[B103-behavsci-15-00350] McVeigh J., MacLachlan M., MacLachlan M., McVeigh J. (2021). Psychological governance and COVID-19: A case study in macropsychology. Macropsychology—A population science for sustainable development goals.

[B104-behavsci-15-00350] McVeigh J., MacLachlan M. (2022). The macropsychology of COVID-19: Psychological governance as pandemic response. American Psychologist.

[B105-behavsci-15-00350] McVeigh J., Mannan H., Ebuenyi I. D., MacLachlan M., Ned L., Velarde M. R., Singh S., Swartz L., Soldatić K. (2024). Inclusive and equitable policies: EquiFrame and EquIPP as frameworks for the analysis of the inclusiveness of policy content and processes. Routledge international handbook of disability and global health.

[B106-behavsci-15-00350] Melton G. B., Melton G. B. (1995). Personal satisfaction and the welfare of families, communities, and society. Nebraska symposium on motivation: Vol. 42. The individual, the family, and social good: Personal fulfilment in times of change.

[B107-behavsci-15-00350] Melton G. B. (2010). In search of the highest attainable standard of mental health for children. Child Welfare: Journal of Policy, Practice, and Program.

[B108-behavsci-15-00350] Mezzina R., Gopikumar V., Jenkins J., Saraceno B., Sashidharan S. P. (2022). Social vulnerability and mental health inequalities in the “syndemic”: Call for action. Frontiers in Psychiatry.

[B109-behavsci-15-00350] Migacheva K. (2015). Searching for puzzle pieces: How (social) psychology can help inform human rights policy. Peace and Conflict: Journal of Peace Psychology.

[B110-behavsci-15-00350] Mitchell T., MacLeod T. (2014). Aboriginal social policy: A critical community mental health issue. Canadian Journal of Community Mental Health.

[B111-behavsci-15-00350] Moreno A., Ardila R., Zervoulis K., Nel J. A., Light E., Chamberl L. (2020). Cross-cultural perspectives of LGBTQ psychology from five different countries: Current state and recommendations. Psychology & Sexuality.

[B112-behavsci-15-00350] Moss S. M., Vollhardt J. R. (2016). ’You can’t give a syringe with unity’: Rwandan responses to the government’s single recategorization policies. Analyses of Social Issues and Public Policy (ASAP).

[B113-behavsci-15-00350] Mowat J. G. (2020). Interrogating the relationship between poverty, attainment and mental health and wellbeing: The importance of social networks and support—A Scottish case study. Cambridge Journal of Education.

[B114-behavsci-15-00350] Mukhtarov F. (2022). Combining behavioural and reflective policy tools for the environment: A scoping review of behavioural public policy literature. Journal of Environmental Planning and Management.

[B115-behavsci-15-00350] Muniz Neto J. S., de Lima A. F., Mir L. L., França L. d. C. (2014). Vigiar e assistir: Reflexões sobre o direito à assistência da ‘adolescência pobre’ = To watch and to attend: Reflections on legal assistance of ‘poor adolescent’. Psicologia em Estudo.

[B116-behavsci-15-00350] Munn Z., Peters M. D. J., Stern C., Tufanaru C., McArthur A., Aromataris E. (2018). Systematic review or scoping review? Guidance for authors when choosing between a systematic or scoping review approach. BMC Medical Research Methodology.

[B117-behavsci-15-00350] Newbigging K., Ridley J. (2018). Epistemic struggles: The role of advocacy in promoting epistemic justice and rights in mental health. Social Science & Medicine.

[B118-behavsci-15-00350] Newbigging K., Ridley J., McKeown M., Machin K., Poursanidou K. (2015). ‘When you haven’t got much of a voice’: An evaluation of the quality of Independent Mental Health Advocate (IMHA) services in England. Health & Social Care in the Community.

[B119-behavsci-15-00350] Newman D., Gordon F. (2021). Leading works in law and social justice.

[B120-behavsci-15-00350] O’Donnell K. S. (2012). Global mental health: A resource primer for exploring the domain. International Perspectives in Psychology: Research, Practice, Consultation.

[B121-behavsci-15-00350] Ogolsky B. G., Monk J. K., Rice T. M., Oswald R. F. (2019). As the states turned: Implications of the changing legal context of same-sex marriage on well-being. Journal of Social and Personal Relationships.

[B122-behavsci-15-00350] OHCHR (Office of the United Nations High Commissioner for Human Rights) (2022). OHCHR and good governance.

[B123-behavsci-15-00350] OPSI (Observatory of Public Sector Innovation) (2021). Behavioural insights units.

[B124-behavsci-15-00350] Oster C., Henderson J., Lawn S., Reed R., Dawson S., Muir-Cochrane E., Fuller J. (2016). Fragmentation in Australian Commonwealth and South Australian State Policy on mental health and older people: A governmentality analysis. Health: An Interdisciplinary Journal for the Social Study of Health, Illness and Medicine.

[B125-behavsci-15-00350] Österman K., Björkqvist K., Wahlbeck K. (2014). Twenty-eight years after the complete ban on the physical punishment of children in Finland: Trends and psychosocial concomitants. Aggressive Behavior.

[B126-behavsci-15-00350] Perucchi J., Rodrigues F., Deotti A., Jardim L. N., Calais L. B. d. (2011). Psicologia e políticas públicas em HIV/AIDS: Algumas reflexões = Psychology and public policy in HIV/AIDS: Some reflections. Psicologia & Sociedade.

[B127-behavsci-15-00350] Petersen I., Evans-Lacko S., Semrau M., Barry M. M., Chisholm D., Gronholm P., Egbe C. O., Thornicroft G. (2016). Promotion, prevention and protection: Interventions at the population- and community-levels for mental, neurological and substance use disorders in low- and middle-income countries. International Journal of Mental Health Systems.

[B128-behavsci-15-00350] Pettigrew T. F. (2011). SPSSI and racial research. Journal of Social Issues.

[B129-behavsci-15-00350] Pinheiro J. d. C., Sousa S. M. G. (2020). Uma Avaliação da Criança como Sujeito Assujeitado no Processo Judicial = An assessment of the child as an objectified subject in the judicial process. Avaliação Psicológica.

[B130-behavsci-15-00350] Premachandra B., Lewis N. A. (2022). Do we report the information that is necessary to give psychology away? A scoping review of the psychological intervention literature 2000–2018. Perspectives on Psychological Science.

[B131-behavsci-15-00350] Pykett J., Jones R., Whitehead M., Pykett J., Jones R., Whitehead M. (2017). Introduction: Psychological governance and public policy. Psychological governance and public policy: Governing the mind, brain and behaviour.

[B132-behavsci-15-00350] Rami F., Searight H. R., Dryjanska L., Battista P. (2022). COVID-19—International psychology’s role in addressing healthcare disparities and ethics in marginalized communities. International Perspectives in Psychology: Research, Practice, Consultation.

[B133-behavsci-15-00350] Riggle E. D. B., Rostosky S. S., Horne S. G. (2010). Psychological distress, well-being, and legal recognition in same-sex couple relationships. Journal of Family Psychology.

[B134-behavsci-15-00350] Ruggeri K. (2017). Editorial: Psychology and policy. Frontiers in Psychology.

[B135-behavsci-15-00350] Salas L. M., Ayón C., Gurrola M. (2013). Estamos traumados: The effect of anti-immigrant sentiment and policies on the mental health of Mexican immigrant families. Journal of Community Psychology.

[B136-behavsci-15-00350] Sales B. D., Krauss D. A. (2015). The psychology of law: Human behavior, legal institutions, and law.

[B137-behavsci-15-00350] Sanchez-Mazas M. (2015). The construction of ‘official outlaws’ Social-psychological and educational implications of a deterrent asylum policy. Frontiers in Psychology.

[B138-behavsci-15-00350] Scanlon C., Adlam J. (2013). Knowing your place and minding your own business: On perverse psychological solutions to the imagined problem of social exclusion. Ethics and Social Welfare.

[B139-behavsci-15-00350] Shand F., Duffy L., Torok M. (2022). Can government responses to unemployment reduce the impact of unemployment on suicide? A systematic review. Crisis: The Journal of Crisis Intervention and Suicide Prevention.

[B140-behavsci-15-00350] Smail D. (1995). Power and the origins of unhappiness: Working with individuals. Journal of Community & Applied Social Psychology.

[B141-behavsci-15-00350] Spears A. P. (2010). The healthy people 2010 outcomes for the care of children with special health care needs: An effective national policy for meeting mental health care needs?. Maternal and Child Health Journal.

[B142-behavsci-15-00350] Steel Z., Momartin S., Silove D., Coello M., Aroche J., Tay K. W. (2011). Two year psychosocial and mental health outcomes for refugees subjected to restrictive or supportive immigration policies. Social Science & Medicine.

[B143-behavsci-15-00350] Thomas G. (2013). A review of thinking and research about inclusive education policy, with suggestions for a new kind of inclusive thinking. British Educational Research Journal.

[B144-behavsci-15-00350] Toquero C. M. D. (2021). Provision of mental health services for people with disabilities in the Philippines amid Coronavirus outbreak. Disability & Society.

[B145-behavsci-15-00350] Torres S. A., Santiago C. D., Walts K. K., Richards M. H. (2018). Immigration policy, practices, and procedures: The impact on the mental health of Mexican and Central American youth and families. American Psychologist.

[B146-behavsci-15-00350] Triana M. d. C., Jayasinghe M., Pieper J. R., Delgado D. M., Li M. (2019). Perceived workplace gender discrimination and employee consequences: A meta-analysis and complementary studies considering country context. Journal of Management.

[B147-behavsci-15-00350] Tricco A. C., Lillie E., Zarin W., O’Brien K. K., Colquhoun H., Levac D., Moher D., Peters M. D., Horsley T., Weeks L., Hempel S. (2018). PRISMA extension for scoping reviews (PRISMA-ScR): Checklist and explanation. Annals of Internal Medicine.

[B148-behavsci-15-00350] Tropp L. (2023). Applying the full force of research and theory to social policy.

[B149-behavsci-15-00350] United Nations General Assembly (2017). Report of the special rapporteur on the right of everyone to the enjoyment of the highest attainable standard of physical and mental health.

[B150-behavsci-15-00350] Uribe Aramburo N. I. (2011). Abuso sexual infantil y administración de justicia en Colombia Reflexiones desde la psicología clínica y forense = Child sexual abuse and the application of justice in Colombia Reflections in clinical psychology and forensics. Pensamiento Psicológico.

[B151-behavsci-15-00350] Valentim J. P. (2013). Brazilian Social Psychology in the international context: A commentary. Estudos de Psicologia.

[B153-behavsci-15-00350] Van Deun H., Van Acker W., Fobé E., Brans M. (2018). Nudging in public policy and public management: A scoping review of the literature. PSA 68th Annual International Conference.

[B152-behavsci-15-00350] Van de Vliert E., Conway L. G., Van Lange P. A. M. (2023). Enriching psychology by zooming out to general mindsets and practices in natural habitats. Perspectives on Psychological Science.

[B154-behavsci-15-00350] Vasquez M. J. T. (2012). Psychology and social justice: Why we do what we do. American Psychologist.

[B155-behavsci-15-00350] Venkatapuram S., Bell R., Marmot M. (2010). The right to sutures: Social epidemiology, human rights, and social justice. Health and Human Rights.

[B156-behavsci-15-00350] Vodanovich S. J., Piotrowski C. (2011). Recent legislation in personnel psychology: An update for I/O practitioners. Organization Development Journal.

[B157-behavsci-15-00350] Ward C., Gale J., Staerklé C., Stuart J. (2018). Immigration and multiculturalism in context: A framework for psychological research. Journal of Social Issues.

[B158-behavsci-15-00350] Watts L., Hodgson D. (2019). Social justice theory and practice for social work.

[B159-behavsci-15-00350] Weissman D. G., Hatzenbuehler M. L., Cikara M., Barch D. M., McLaughlin K. A. (2023). State-level macro-economic factors moderate the association of low income with brain structure and mental health in U.S. children. Nature Communications.

[B160-behavsci-15-00350] West A. E., Williams E., Suzukovich E., Strangeman K., Novins D. (2012). A mental health needs assessment of urban American Indian youth and families. American Journal of Community Psychology.

[B161-behavsci-15-00350] Whelan D. (2021). Application of the paternalism principle to constitutional rights: Mental health case-law in Ireland. European Journal of Health Law.

[B162-behavsci-15-00350] Wolf S., Aber J. L., Morris P. A. (2013). Drawing on psychological theory to understand and improve antipoverty policies: The case of conditional cash transfers. Psychology, Public Policy, and Law.

[B163-behavsci-15-00350] Wong D. F. K., Zhuang X. Y., Pan J. Y., He X. S. (2014). A critical review of mental health and mental health-related policies in China: More actions required. International Journal of Social Welfare.

[B164-behavsci-15-00350] Woodford M. R., Kulick A., Garvey J. C., Sinco B. R., Hong J. S. (2018). LGBTQ policies and resources on campus and the experiences and psychological well-being of sexual minority college students: Advancing research on structural inclusion. Psychology of Sexual Orientation and Gender Diversity.

[B165-behavsci-15-00350] World Health Organisation (WHO), WHO Commission on Social Determinants of Health (2008). Closing the gap in a generation: Health equity through action on the social determinants of health.

[B166-behavsci-15-00350] Ximenes V. M., Cidade E. C., Nepomuceno B. B. (2015). Psicología comunitaria y expresiones psicosociales de la pobreza: Contribuciones para la intervención en políticas públicas = Community psychology and psychosocial expressions of poverty: Contributions for public policy intervention. Universitas Psychologica.

[B167-behavsci-15-00350] Yadegarfard M., Bahramabadian F. (2014). Sexual orientation and human rights in the Ethics Code of the Psychology and Counseling Organization of the Islamic Republic of Iran (PCOIRI). Ethics & Behavior.

[B168-behavsci-15-00350] Zayas L. H., Bradlee M. H. (2014). Exiling children, creating orphans: When immigration policies hurt citizens. Social Work.

