# Supplementary material for: Macropsychology: A Systematic Scoping Review of the Psychology Literature on Public Policy and Law
Source: Behav Sci (Basel). 2025 Mar 12;15(3):350. doi: 10.3390/bs15030350 (PMC11939743; doi:10.3390/bs15030350)
Supplement: Supplementary file 1 [file behavsci-15-00350-s001.zip › Supplementary File S2_Data Extraction Sheet.pdf]

Macropsychology: A Scoping Review of the Psychology Literature on Public Policy and Law

Supplementary File S2: *Data Extraction Sheet*

1) Empirical Articles

Article No:	Authors:	Aims:	Design:	Population of Interest:	Geographical Location:	Main Findings:	Main Conclusions:	Key Words / Topics:
2.	Callaghan et al. (2017)	To analyse Child and Adolescent Mental Health Services (CAMHS) policies with regards to how childhood, mental health, and CAMHS are constituted.	Policy analysis. 15 policy documents were analysed (9 policies pre-2010 and 6 policies post 2010).	Children and adolescents.	England.	The authors identify how distress experienced by children is individualised, via medicalising discourses and shifting understandings of the relationship between socioeconomic context and mental health. This is evidenced in a transition from viewing children's mental health problems as determined by socio- economic inequities, to a view that children's mental health should be addressed early to mitigate against future socio-economic burdens.	The authors examine how concepts of 'parity of esteem' and 'stigma reduction' may inadvertently increase the individualisation of children's mental health problems.	Camhs, Child and Adolescent Mental Health, Health Inequalities, Medicalisation, Parity of Esteem, Policy.
3.	West et al. (2012)	To conduct a qualitative study of community mental health needs amongst an urban population of American Indian youth, conducted as part	Focus groups.	107 urban American Indian youth and families.	United States.	Findings indicated numerous community characteristics, mental health and wellness needs, and service system needs with regards to developing a system of care. Key community, cultural, and social processes were also identified, indicating the	Systems/policy changes are needed to support systems of care that address the needs of communities and reflect key values of the SOC philosophy. The findings indicate: (1) support for an ecological focus - a	American Indian, Urban, Children and Adolescents, Mental Health, System of

		of the planning process for a system of care (SOC).				importance of broader system changes to promote a sustainable system of care.	transition away from a sole or primary focus on clinical services, to address a wider range of programmes and services for at-risk youth and families (such as community stressors, poverty, unemployment) with funding to support prevention and the strengthening of informal supports for families; and (2) support for a community-based, family-centred care approach.	Care, Qualitative.
4.	Ogolsky et al. (2019)	To examine changes in personal well-being among individuals in same-sex relationships throughout the transition to federal marriage recognition.	Quantitative survey (Longitudinal panel data were collected once before and at three time points after the U.S. Supreme Court decision in	279 individuals in same-sex relationships.	United States.	Before the ruling, levels of internalized homonegativity, isolation, and vicarious trauma were positively associated with psychological distress. Levels of felt stigma and vicarious trauma were negatively associated with life satisfaction. After the ruling, trajectories of psychological distress decreased over time for people who experienced higher (vs. lower) initial levels of internalized homonegativity, isolation, and vicarious trauma. Trajectories of life satisfaction	The findings indicate that the *Obergefell vs. Hodges* decision providing legal recognition of same-sex marriage in the U.S. had a demonstrable impact on experiences of minority stress and well-being. The legal change had the strongest impact on individuals who needed it the most: individuals who experienced the highest levels of minority stress in the form of internalized	Discrimination, Family Policy, Family Relations, Hdfs, LGBTQ, Minority Stress, Psychology, Same-Sex Marriage, Satisfaction, Stress, Well-Being.

			the *Obergefell vs. Hodges* case, which led to federal recognition of same-sex marriage).			increased over time for individuals who experienced higher (vs. lower) initial levels of vicarious trauma. These changes occurred over and above the effects of state and local recognition and marital status.	homonegativity, isolation, and vicarious trauma.	
5.	Shand et al. (2022)	To conduct a systematic review to examine whether government policies aimed at addressing unemployment can moderate the effects of unemployment on suicide and self-harm.	Systematic review.	People who are unemployed.	Not specified.	Six studies were identified. Each study used an ecological design and suicide deaths as the outcome. Three of five studies examining unemployment benefits reported a negative association with suicide rates. Studies exploring the effects of active unemployment policy and employment protection legislation reported evidence of beneficial effects. The effects of the policies were small and had particular benefit in decreasing suicide rates among men.	Results of the review indicate that unemployment policies can mitigate the relationship between unemployment and suicide, particularly amongst men.	Suicide Prevention, Welfare Policies, Unemployment Benefits, Public Health, Mental Health.
6.	Lund et al. (2011)	To explore the key challenges in the South African mental health system, to provide	Qualitative (Interviews and	99 interviews and 12 focus groups with	South Africa.	Key challenges that were identified included: the lack of officially endorsed mental health policy; the continued low priority of mental health;	Recommendations include formulating an overarching national mental health policy; lobbying for mental health	Mental Health, Healthcare Systems, South Africa,

		a greater understanding of these challenges and to outline stakeholders' recommendations on the most effective way to address them.	focus groups).	mental health care system stakeholder s.		limited intersectoral policy integration; stigma and discrimination; insufficient integration of mental healthcare with primary healthcare; and 'de-hospitalisation' instead of 'de-institutionalization'.	on national policy agendas; incorporating mental health in poverty alleviation programmes; more effective educational and awareness-raising campaigns; standardised evidence-based training of health personnel; and the development of community-based mental healthcare.	Health Policy, Health Priorities.
7.	Bielsa et al. (2010)	To assess and map Child and Adolescents' Mental Health (CAMH) infrastructures, policies, and programs across 15 European countries using a standardized questionnaire; and to provide an overview of European findings, offer recommendations, and critically discuss the methodology and	Mixed methods (questionn aire and workshops /round-table discussion).	Children and adolescents.	Belgium, Bulgaria, Estonia, Finland, Germany, Greece, Hungary, Latvia, Lithuania, Norway, Poland, Romania, Spain, Slovenia, and England.	As indicated by the findings, 1) the implementation and outcomes of CAMH policies and action plans are not evaluated systematically in Europe, 2) prevalence data on positive mental health, mental disorders and risk, and vulnerable populations is heterogeneous and patchy across Europe, 3) children and adolescents are seldom involved in decision-making processes influencing CAMH practices, 4) CAMH issues are not included in all relevant higher education qualifications, 5) budgets dedicated to CAMH issues are not identifiable, specific or adequate in the	Several recommendations are made, including improved policy evaluation; strengthened monitoring and assessment of trends in CAMH; greater youth involvement; improved training and capacity; sufficient budgets for CAMH; and improved mental health understanding.	Mental Health, Promotion, Prevention, Children, Adolescents.

		achievements of the CAMHEE WP4 study.				long-term, and 6) stakeholders and the general public are not widely aware of the determinants of CAMH.		
8.	Bjerge et al. (2020)	To assess policies in the fields of drug use and treatment, unemployment, and mental health separately, and to collate the findings to conduct a cross-sectorial analysis of how and why individuals with a mix of problems related to the three fields are produced into particular 'kinds' of subjects amenable to particular kind of tools, interventions and outcomes.	Policy analysis.	Drug users enrolled in drug treatment and mental health services who receive unemployment benefits.	Denmark.	The findings demonstrate how policies problematize drug users with complex problems as both morally obliged to find inner motivation and willpower and as victims of deprivation, hindering them from the benefits of leading a 'normal' life.	Although it is outlined in all policies that multiple factors may impact individuals (such as drug use, unemployment, and mental health), the fixing of it is mainly forecast as the outcome of causal links related to one particular kind of problem, including particular ways of intervening, rather than as being affected by numerous forces, structures, institutions, or people.	Drug Use and Treatment, Unemployment, Mental Health, Policy Analysis.
9.	Grzanka, DeVore, et al. (2020)	To understand sexual and gender minority individuals’	Interviews.	20 sexual and gender minority (SGM)	United States.	The participants viewed the law as discriminatory, although they perceived the conscience clause as legalizing	The authors situate the research findings in the context of research on structural stigma. They	Mental Health Care, Discrimination, Law,

		experiences with mental health services and their view of the law that permits counsellors and therapists to deny services to a client based on the practitioner's "sincerely held principles" (the "conscience clause").		individuals living in Tennessee.		discrimination against all stigmatised groups—not just SGM individuals.	argue for counselling psychologists to actively engage in combating conscience clauses, which may have a significant impact on the engagement of stigmatised and vulnerable populations with mental health services.	Conscience Clause, Ethics.
10.	Newbigging & Ridley (2018)	To explore a new approach to theorising advocacy based on Fricker's evolving concept of epistemic injustice and grounded in empirical data from mental health service user accounts of statutory advocacy; and to identify the implications for promoting rights	Interviews and focus groups.	90 interviews with people subject to detention, and 3 focus groups with mental health service users.	England.	The findings indicate that advocacy can serve both to increase re-cognition and legitimation of people detained under mental health law as' epistemic agents' and go some way to promoting testimonial justice through strengthening voice and addressing prejudicial-credibility deficits; While advocacy is having little impact on hermeneutical injustice in enabling people to reclaim or develop different understanding of their experience, the advocacy role extends to acting as an epistemic witness and ensure	The concept of epistemic injustice provides a valuable theoretical basis for understanding the worth of advocacy in addressing testimonial injustice as well as its relative weakness in overcoming hermeneutical injustice. The challenge of independent advocacy to the dominant discourse within mental health is considered and questions raised about the place of advocacy in modern democratic mental health systems.	England, Advocacy, Mental Health, Mental Illness, Epistemic, Injustice, Social Justice, Human Rights, Mental Health Law.

		under mental health legislation and achieving greater democracy in mental health services.				that epistemic agency is protected.		
11.	Li & Hui (2020)	This paper aims to disclose the effect of government policies on mental health of left-behind children (LBC) in China.	Survey.	Left-behind children from 10 rural primary schools in remote areas of western China.	China.	The results indicate that the LBC's were significantly higher than non-LBC in physical symptom only, and slightly higher in the other respects; the female LBC whose father is migrant worker had much worse mental health than their male counterparts in learning, anxiety, social anxiety and self-blame, allergic and terrorist tendencies; male LBC had much higher anxiety of physical symptom than female LBC.	The article concludes that the government policies have effectively improved the mental health of the LBC, and special care should be given to female LBCs whose father is migrant worker. The research results provide the direction for LBC policy-making.	Mental Health Diagnostic Test (Mht), Left-Behind Children (Lbc), Government Policies, Migrant Worker
12.	Salas et al. (2013)	To examine the effects of immigration legisl ation on Mexican immigrant families' health and mental health.	Focus groups.	Focus groups with youth and adults who were immigrants or children of immigrants (n = 43).	United States.	Participants shared the risky conditions that are part of their everyday life and their feelings of isolation, powerlessness, frustration, fear, stress and chronic trauma they experience with major themes arising: (a) risking their lives (dangers involved in migrating to the United States); (b) looking out	Participants' stories conveyed in this study run counter to dominant narratives of Mexican immigrants as criminals. In addition, the families encounter contradictory policies and practices; including a formal political system that does not	Family, Immigration, Mental Health, Mexican Americans, Health Care Policy.

						the window (living in a constant state of fear, of being arrested and deported; significantly restricting how they live their lives); and (c) They are traumatized (effects on children, how women's stress is manifested, and men's feelings of powerlessness).	support undocumented entry and an informal system that recruits undocumented workers and benefits from their labour. These findings indicate that immigrant families are in much need for services and advocacy efforts are needed to support access to care as well. The article suggests that the dominant frame labelling undocumented Mexicans as "illegal immigrants" to be considered under a new frame of "trauma survivor".	
13.	Kaczkowski et al. (2022)	To examine the association of lesbian, gay, bisexual, transgender, and questioning (LGBTQ)-supportive school policies an d practices with psychosocial health outcomes among lesbian,	Survey.	The 2014 and 2016 School Health Profiles data from principals and health educators from 117 schools examined LGBTQ-	United States.	Several LGBTQ-supportive school policies and practices were significantly associated with lower odds of feeling threatened at school, suicide-related behaviours, and illicit drug use among LGB students; in addition having a gay-straight alliance or similar club was linked to multiple health outcomes for heterosexual students, whereas other policies and practices were	These findings suggest that LGBTQ-supportive school policies and practices are significantly associated with improved psychosocial health outcomes among both LGB and heterosexual students.	High-Risk Substance Use, Lgb Youth, Lgbtq-Supportive School Policies, Mental Health and Suicide, Student Health

		gay, bisexual (LGB), and heterosexual students.		supportive school policies and practices. The 2015 and 2017 Youth Risk Behavior Survey data from 75,638 students from the same schools examined psychosocial health outcomes.		significantly associated with lower odds of safety concerns at school, forced sexual intercourse, feeling sad or hopeless, and illicit drug use; increased sum of policies and practices was linked to lower odds of suicide-related behaviours among LGB students and safety concerns and illicit drug use among heterosexual students.		Disparities, Violence Victimization
14.	Oster et al. (2016)	To describe how older people's mental health is governed through policy discourse by examining Australian Commonwealth and South Australian State government policy documents, including commentaries	Review of policies and commentaries.	Older people.	Australia.	Three policy approaches were identified where older people are (1) absent from policy, (2) governed as responsible, active citizens or (3) governed as passive recipients of health care.	The fragmented policy response to older people's mental health reflects fragmentation in the Australian policy environment. It constructs an ambiguous place for older people within neo-liberal governmental rationality, with significant effects on the health system, older people, and their carers.	Discourse, Governmentality, Mental Health, Older People, Policy.

		from professional groups, advocacy groups and non-governmental organisations.						
15.	Abreu et al. (2021)	To explore parental figures' reactions and coping strategies with recent proposed and enacted laws and bills in the United States restricting access to gender-affirming healthcare for trans and gender diverse (TGD) youth.	Qualitative survey.	138 parental figures of trans and gender diverse youth who currently or previously lived in the US.	United States.	Thematic analysis revealed four themes depicting participants' cognitive reactions, including: (a) violation of rights, (b) increased stigma, (c) decreased quality of healthcare, and (d) support for child's journey; three themes emerged about participants' emotional reactions, including: (a) fear and anxiety, (b) anger, and (c) relief; additionally participants shared narratives about how they are coping with these antitransgender laws and bills, included: (a) activism and advocacy, (b) educating others, (c) seeking support from communities/groups, and (d) relocation and avoidance.	Participants viewed these oppressive antitransgender laws and bills as a violation of their and their child's rights, a source of increased stigma toward their TGD child and the TGD community overall, and as having a negative impact on the quality of healthcare for TGD people. This study provides insight about how these new attacks on TGD youth are affecting their caregivers by specifically uncovering how oppressive political environments are yet another system that negatively affects parental figures of TGD youth.	Parents, Mental Health, Transgender Youth, Transgender Health, Legislation
16.	Hatzenbuehler et al. (2017)	To examine associations between the state-level policy climate related to	The authors formulated a multi-sectoral	Latinos.	United States.	The study found robust individual- and state-level confounders with individuals in states with a more exclusionary immigration	These results suggest that restrictive immigration policies may be detrimental to the mental health of Latinos in the	United States, Immigration Policies, Latino

		immigration and mental health outcomes among Latinos.	policy climate index. They then examined the relation of this policy climate index to two mental health outcomes among Latinos from 31 states in the 2012 Behavioral Risk Factor Surveillance System.			policy climate having higher rates of poor mental health days than participants in states with a less exclusionary policy climate (RR: 1.05, 95% CI: 1.00, 1.10) and a significantly higher association among Latinos versus non-Latinos (RR for interaction term: 1.03, 95% CI: 1.01, 1.06); in addition Latinos in states with a more exclusionary policy climate had 1.14 (95% CI: 1.04, 1.25) times the rate of poor mental health days than Latinos in states with a less exclusionary policy climate.	United States. This study provides an important contribution to our understanding of the mental health consequences of exposure to laws that marginalize and discriminate against Latinos by providing some of the first evidence to suggest that the current policy environment surrounding Latinos may be adversely affecting the mental health of this population.	Health, Discrimination, Stigma, Mental Health, Social Determinants
17.	Abreu et al. (2022)	To explore parental figures' perceptions of how bans on gender affirming care affect their TGD child and parental figures'	Qualitative survey.	134 self-identified parental figures of transgender and gender diverse youth.	United States.	The impact that these antitransgender laws and bills have on TGD youth, include 5 themes: (a) depression and suicidal ideation/risk of suicide, (b) anxiety, (c) increased gender dysphoria, (d) decreased safety and increased	This study sheds light on different ways (i.e. increased depression, suicidal ideation, anxiety, stigma, and gender dysphoria, decreased safety, and lack of access to medical care) in which	Transgender, Youth, Mental Health, Antitransgender Legislation,

		advice for legislators/policy-makers regarding the impact of these laws and bills on the well-being of TGD youth.				stigma, and (e) lack of access to medical care. Feedback from parental figures to legislators/policy-makers regarding the impact of these laws and bills included: (a) transgender youth health is not a political issue, (b) decriminalize gender affirming medical care, (c) decrease discrimination and violence against transgender people, and (d) become educated on transgender health-care issues.	TGD youth are being affected by these bills. This article extends a call to mental health practitioners to engage individual and systemic interventions in order to provide support and advocate for TGD youth and their families by not making transgender health a political issue, decriminalizing gender-affirming medical care, decreasing discrimination and violence against transgender people, and educating themselves on transgender health-care issues.	Parental Figures.
18.	Chen (2020)	This paper aims to improve the mental health of labourers through the publicity of social security law.	Quantitative survey.	Labourers.	China.	This study found that comprehensive coverage is the ultimate goal of the publicity of social security law and that the female labourers have more serious mental health problems than male labourers, but their mental health is greatly improved after the publicity of social security laws.	The study concludes that the social security law of labourers includes the social insurance legal system, social security fund pooling system, and household registration reform system, etc., all of which involve the labourers' own legal rights and interests, and must comply with the principles	Social Security Law, Rights and Interests, Labourers, Mental Health

							of sustainable development, stability, fairness and efficiency. With the publicity of social security law having a significant effect on improving the mental health of labourers, especially female labourers, the mental health of labourers at all ages has improved significantly.	
19.	Gómez (2010)	To examine the situation in Colombia regarding the rights of persons with mental disorders and to understand judicial reasoning in this area, as a means of obtaining tools for the daily work in mental health.	Review of 35 judgments of the Constitutional Court referring to the *tutela actions* in relation to mental health.	Persons with mental health issues.	Colombia.	This study finds that most of the guardianship actions related to mental health are demanded by people with mental disorders or by their relatives; also showing that the issues most frequently demanded are health institutions and the rights requested are restitution of life, dignity, and health.	The article concludes that the knowledge and analysis of the judgments of the Court help to open more possibilities of action to mental health professionals, who can use that judicial experience in understanding and handling their own cases.	Laws, Mental Health, Patient Rights.
20.	Woodford et al. (2018)	To investigate the association between campus-based structural factors and the	Quantitative survey and objective data.	Sexual minority college students (study	United States.	The study finds that the non-discrimination policies inclusive of gender identity and sexual orientation (vs. only sexual orientation), offering at	The results highlight the importance of particular structural initiatives on campus in protecting LGBQ+ collegians from	Minority Stress, Structural Stigma/Inclusion, Mental

		experiences and psychological well-being of cisgender LGBQ+ college students.		sample: 268 participants from 58 colleges).		least one for-credit LGBTQ course, and a higher ratio of LGBTQ student organizations to the student population were directly associated with participants reporting lower levels of discrimination, which was associated with less distress and higher self-acceptance.	discrimination in addition to showing the value of studying specific structural initiatives when investigating structural stigma and inclusion.	Health, Policy, Campus Climate.
21.	Campbell & Steel (2015)	To explore the relationship between mental wellbeing, living conditions, and Australia's detention policies in light of human rights by studying the experiences of asylum seekers in Australia.	Qualitative: Observations, interviews, and document analysis.	7 asylum seekers and 3 professionals working with them.	Australia.	The findings highlight a discrepancy between asylum seekers' experiences under immigration detention policy and Australia's human rights obligations. This paper reports on the mental distress suffered by asylum seekers in detention, the environments of constraint in which they live, and aspects of detention centre policy that contribute to these environments indicating human rights violations for asylum seekers in detention in Australia.	The authors make considerations of alternatives to detention as well as improving detention centre conditions and suggest that the World Health Organization's Quality Rights Tool Kit might provide the basis for a framework to review Australia's immigration detention system with particular focus on the poor mental wellbeing of asylum seekers in detention.	Wellbeing, Policy, Asylum Seekers, Human Rights, Mental Health.
22.	Major (2018)	To test whether changing the way stories frame depression affects how audience	Experimental study (2×2 between-groups	People with depression.	United States.	Results suggest that Positive or negative emotional responses do not play a role in the framing effects on attribution of societal or individual	To gain public support for policies that address mental health issues, authors suggest that we must achieve a much	Mental Health News | Support Policy | Civic Engagement

		members attribute responsibility for depression and their intentions to participate in civic engagement activities supporting policy solutions.	factorial design).			responsibility, except for civic engagement intentions. Both positive and negative emotional responses were significant with the findings warranting more investigation to determine a mediating effect.	greater understanding of the public's response to the news frames used to present the policies. While the current research is a step in that direction, a "one size fits all "framing strategy for health issues and health policy will not work as the best answer may be to examine the effects of generic frames representing a more real-world journalistically valid context in which to conduct this research, across health issues and policies to deter-mine the effects including significant interactions.	Intentions | Public Health | Depression | Community Involvement | Health Promotion | Mental Health | News Media | Public Health | Depression (Emotion)
23.	Gerlinger et al. (2019)	To analyse and compare the results of the revision processes of the accommodation laws or laws of the states providing assistance for the mentally ill in order to define the	Analysis of the revision processes of mental health law.	People with severe mental health issues.	Germany.	Despite the revisions, the state laws are extremely heterogeneous and, in many states, do not fully comply with the requirements of the United Nations Convention on the Rights of Persons with Disabilities (UN-CRPD) or the highest courts' decisions.	The authors conclude that the state laws should be harmonized, particularly where they restrict basic and human rights, e. g. regarding prerequisites and objectives of involuntary commitment and coercive measures.	Accommodat ion, Help for the Mentally Ill, Laws, Precautions, Coercion

		need for action for federal and state legislature.						
24.	Triana et al. (2019)	To examine how the context influences the relationship between employees' perceptions of gender discrimination and outcomes at work; drawing on relative deprivation theory.	Meta analysis. Complementary Studies.	Employees experiencing gender discrimination.	International.	Findings show that perceived gender discrimination is negatively related to job attitudes, physical health outcomes and behaviours, psychological health, and work-related outcomes (job-based and relationship-based) with stronger correlations in countries with more broadly integrated labour policies and stringently enforced labour practices focused on promoting gender equality, and in countries with more gender-egalitarian cultural practices.	Complementary studies supported the meta-analytic findings and provided evidence of the relative deprivation rationale with implications for research and practice including the need to consider the influence of the country context in organizational decisions to prevent and address gender discrimination and its consequences for employees and ultimately, for employers.	Relative Deprivation Theory, Gender Discrimination, Job Attitudes, Physical Health Outcomes and Behaviours, Psychological Health, Job-Related Outcomes, Country Context, Culture
25.	Browne et al. (2020)	To examine the influences, processes, skills, and knowledge underpinning policy work by clinical psychologists, and the challenges and	Interviews.	37 clinical psychologists.	UK.	The analysis yielded six themes, grouped into two domains: (a) 'getting there,' describing participants' professional journeys to policy work, including early influences and career paths; and (b) 'being there' describing their experiences of working in this way, the challenges and	Clinical psychologists already possess core clinical and research skills that may be adapted to work within broader political systems. However, they need to learn to use their existing skills in a different context, and also acquire	Policy, Social Action, Clinical Psychology, Public Health, Psychologists' Roles

		facilitators encountered.				facilitators in the process, and the skills and knowledge upon which they drew.	some additional skills unique to policy-level work	
26.	Baker & Brassard (2019)	To address 3 sets of questions using the most current State data and examining State statutes as to how they were defining Psychological Maltreatment (PM). 1. To address variability in reported rates of Psychological Maltreatment (PM) as compared to reported rates of other forms of child maltreatment. 2. How PM was addressed in State statutes. 3. The relationship between NCANDS data and elements of the State statutes.	US State statutes were coded and compared with rates of types of child maltreatm ent in National Child Abuse and Neglect Data System data sets.	Children.	United States.	Study found difference in reported rates of PM between the State with the lowest rate and the State with the highest rate (in 2014) was 523-fold, much higher than for physical (30-fold) and sexual abuse (20-fold) but not neglect (524-fold). 2. Statutes still use the term 'mental injury' from the original Child Abuse Prevention and Treatment Act 1974, with two thirds not defining it. 3. Reported rates of PM in NCANDS were not correlated with whether PM was defined in the statute but when a harm standard was present, reported rates were statistically lower. Almost 70% of statutes mentioned a current trend (e.g., sexual /human trafficking) showing a willingness by States to amend statutes.	A common, reliable definition of PM (and other forms of maltreatment) in CAPTA, NCANDS, and US State statutes is necessary for the US to have a surveillance system that allows for the assessment of the effects of policies on reported rates of all forms of maltreatment.	Psychologica l Maltreatment , Emotional Abuse, Emotional Neglect, State Statutes, Child Maltreatment Definitions

28.	Ximenes et al. (2015)	To analyse from the Community Psychology's perspective, psychosocial expressions of poverty and their contributions for intervention in public policy.	Mixed methods (quantitative and qualitative).	417 adult participants living in a rural and urban community.	Brazil.	Study finds that poverty involves moral explanations about the poor: Family, neighbours and religion are emphasized as sources of support, which highlights the need for strategies, community relations and resizing of performance in public policy.	This study suggest that it is evident that Brazilian social assistance policies stand out as a point of reference for intervention and must be assessed as a protective element against adversities coming from contexts of poverty; highlighting the need for strategies to strengthen community relations and the dimensioning of intervention in public policies; with Community psychology leaning towards subjective expressions of poverty and strengthening the performance of public policies and community support networks.	Community Psychology, Poverty, Public Politics, Subjectivity, Social Support.
29.	Furlan & Pelissari (2013)	To discuss a survey that aimed to identify the issues and the contexts in the journal Psychology and Society and in the proceedings of the National	Survey (to examine the issues and contexts in the journal Psychology and Society and in the	Not specified.	Not specified.	Socio-political-cultural contexts and social policies and projects, in which the first reveals a high incidence and the second, low incidence in publications are discussed. The data suggest a gradual increase, from one year to the next, as can be seen in the productions that are inserted and that reflect	These data reveal the commitment of psychology in entering the spaces for the construction of public policies and social projects in order to ensure the promotion and defence of human rights and a society in which it is worth living, i.e., interest	Social Project, Social Policy, Psychology, Human Rights.

		Meetings of ABRAPSO, both published by the association, which cover the period from 2000 to 2010.	proceedings of the National Meetings of ABRAPSO).			on the context of social policies and projects.	in the construction of the common welfare.	
30.	Girvan & Marek (2016)	To propose a basic organizing framework for when a plaintiff's demographic information. may impact civil jury awards, accounting for psychological and structural sources of bias and the ways in which they may interact when jurors have more or less discretion; and to employ a methodological innovation to overcome the absence of plaintiff demographic information.	Quantitative (the data set comprised of jury verdicts in tort cases, alongside information from the US Census Bureau in relation to race and ethnicity).	Jurors and plaintiffs.	United States.	The results were consistent with the psycho-structural framework: where jurors had discretion, they awarded less to black plaintiffs than to white plaintiffs, indicating potential psychological bias. Where jurors had little discretion, they awarded less to female plaintiffs and more to Asian plaintiffs than to male and white plaintiffs, respectively, a potential reflection of structural income disparities.	This study introduces and provides a conceptual test of a novel framework for when bias is most likely to impact damage awards due to race and gender bias in civil cases; the framework and method seem promising for exploring relationships between structural and psychological bias and differential civil jury awards using advances in social demography.	Discrimination, Demography, Jury Decision-Making, Psychology and Law, Race, Ethnic, And Gender Bias, Tort Damage Awards

31.	de Freitas et al. (2018)	To present a systematic review and a meta-analysis of the correlates of perceived ethnic discrimination among ethnic minority persons living in European countries.	Systematic review and meta-analysis.	Ethnic minority persons.	Finland; France; Germany; Greece; United Kingdom; Netherlands; Norway; Portugal; Spain; and Switzerland.	The study finds discrimination positively associated with symptoms of psychiatric disturbances, depression, psychosis, perceived stress, and externalizing Behavior; In addition, discrimination is negatively associated with the self-esteem, positive evaluation of life, self-efficacy/mastery, well-being, and psychological adaptation of migrants. The results suggest that the strength of association is moderated by persons' gender, age, and ethnicity and countries' multicultural preference and countries' rating in the Migrant Integration Policies Index (MIPEX), in regard to labour market mobility, permanent residence, and antidiscrimination policies in some cases.	The results show that fostering multiculturalism, and implementing broad antidiscrimination policies has a protective function in the face of discrimination to mitigate the detrimental effect of discrimination on many psychosocial dimensions. The article highlights the need for governmental agencies to develop systemic and concrete interventions to decrease ethnic prejudice in Europe by enforcing the implementation of these policies to promote the psychological adjustment of ethnic minority persons.	Discrimination, Ethnic Minority, Europe, Psychological Adjustment, Migrants' Integration Policies.
32.	Riggle et al. (2010)	To examine the associations between legal recognition and psychological distress or well-being of couple members in same-	Quantitative survey.	Same-sex couples.	United States.	Study round that participants in committed or legally recognized relationships reported less psychological distress (i.e., internalized homophobia, depressive symptoms, and stress) and more well-being (i.e., the	The authors propose that Legal relationship recognition does not eliminate minority stress nor stigmatization of a LGB or same-sex partner identity; however, the findings suggest that legal	Lesbian/Gay, Same-Sex Couples, Marriage, Minority Stress, Relationship Commitment

		sex couple relationships.				presence of meaning in life) compared to single participants; in addition significant group differences indicated that participants in a legally recognized relationship reported less internalized homophobia, fewer depressive symptoms, lower levels of stress, and more meaning in their lives than those in committed relationships, even after controlling for other factors.	recognition is an important macro-environmental factor that may affect the psychological health and well-being of same-sex couple members.	
33.	Elgar et al. (2021)	To investigate the impact of relative food insecurity on mental health and life satisfaction within countries, considering absolute food insecurity differences and to explore the strength of associations in nations with lower prevalence of food insecurity.	Quantitative survey data from the Gallup World Poll collected in 160 countries and a measure of relative deprivation (Yitzhaki index).	People experiencing relative food insecurity.	International: including 160 countries.	Study found that relative food insecurity—based on national or regional reference groups—related to more mental health symptoms, lower positive wellbeing, and lower life satisfaction after controlling for absolute food insecurity, household income, and country differences. The analysis also found that relative food insecurity was more strongly related to mental health and wellbeing where the prevalence of food insecurity was lower.	In agreement with relative deprivation theory, individuals who live with constant worries about not getting enough food, have to skip meals, or face chronic hunger are deprived of material and social resources that support mental health and wellbeing, especially in settings where food insecurity is less common and potentially more stigmatised. The findings highlight the negative health consequences of material deprivation and	Food Insecurity | Mental Health | Well Being | Life Satisfaction | Global Food Policy | Reference Groups | Health Care Policy Cross Cultural Differences | Social Deprivation | Surveys | Food

							unfavourable social comparisons.	
34.	Grzanka, Spengler et al. (2020)	To assess the LGBT+ Tennesseans' perceptions of the law and how it may affect their help-seeking attitudes and behaviours.	Quantitative survey.	LGBT+ individuals (n = 346).	United States.	The study indicates widespread awareness of the law among the LGBT+ respondents and deep scepticism toward mental health care, in addition to viewing the law as a cover for discrimination.	The authors stress the need for broader research on conscience clauses and call for advocacy against these laws, which have the potential to engender widespread harm to multiple minority groups.	Mental Health Care, Discrimination, Law, Conscience Clause, Ethics
35.	Kourgiantakis et al. (2022)	To explore the literature on recovery policies in mental health and addictions from a social work perspective.	Scoping review.	Not specified.	Not specified.	The results suggest a need to strengthen training for social workers on recovery, increase the focus on policies for addiction recovery, reduce coercive treatment policies, support families, and reduce stigma and discrimination through equitable policies.	The authors highlight the importance of policies that implement recovery principles and aim to eradicate stigma, racism, and discrimination for individuals with mental health and addiction concerns and their families. Service users' and communities' approaches to healing, wellness, and recovery promoting recovery should be taken as incorporated into the development of policies and in ensuring that policies are adequately implemented in service settings.	Policy, Recovery, Mental Health, Addiction, Social Work, Scoping Review.

36.	Kleintjes et al. (2012)	To document the views of South African mental health care service users on policy directions and service developments that are required to support their recovery.	Interviews.	Interviews were conducted with 40 service users and service user advocates.	South Africa.	Service user priorities included addressing stigma, discrimination and disempowerment, and the links between mental health and poverty. Suggestions from service users included: that these challenges be addressed through public awareness campaigns, legislative and policy reform for rights protection, development of a national lobby to advocate for changes, and user empowerment which can be facilitated through opportunities for improved social relatedness and equitable access to social and economic resources.	This study suggests three strategies to bridge the gap between mental health care service users' rights and needs and unsupportive attitudes, policies and practice: 1. giving priority to service user involvement in policy and service reform, 2. creating empathic alliances to promote user priorities, and 3. building enabling partnerships to affect these priorities.	Mental Health, Policy, Participation, South Africa
37.	Spears (2010)	To assess the effectiveness of the Maternal and Child Health Bureau's (MCHB) Six Core Outcomes for children with special health care needs (CSHCN) as indicators in	Quantitative analysis of data from the 2001 National Survey of Children with Special Health Care	Children with special health care needs.	United States.	This study considered eight Outcomes and sub-categories of Outcomes and found that one fifth of the 2.3 million CSHCN in the U.S. who needed mental health care in 2001 did not receive all of the mental health services that they needed. Sixty-one percent of CSHCN with a need for mental health care had care that fulfils six of the eight considered	The initial estimates generated by this study indicate that the MCHB Outcomes are important in meeting children's mental health needs and are important indicators for informing MCHB policy with the authors suggesting that efforts can be made to improve access for CSHCN.	Children With Special Health Care Needs, Children, Access, Medical Home, Mental Health, Maternal and

		measuring the degree to which mental health care needs are met.	Needs for 9,748 children with special healthcare needs who needed mental health care.			Outcomes. Logistic analysis indicates that individual fulfilment of each of the Core Outcomes and fulfilment of additional Outcomes have a significant association with reducing the probability of having an unmet mental health care need for CSHCN.		Child Health Bureau
38.	Fullen et al. (2019)	To explore licensed professional counsellors' experiences of turning away Medicare beneficiaries because of the current Medicare mental health policy through interpretative phenomenologica l analysis.	Qualitativ e interviews.	Licensed professional counsellors (n = 9).	United States.	The results suggest that licensed professional counsellors' experiences indicate that current Medicare provider regulations interfere with mental health care accessibility and availability for Medicare-insured populations.	The authors highlight an existing gap in the administration of Medicare services for clients seeking counselling treatment for mental health conditions, and have attempted to illuminate how current policy impacts the Medicare-insured, as well as LPCs who are involved in their mental health care; and suggest that future revisions to Medicare policy allowing for the inclusion of LPCs to provide counselling treatment to Medicare-insured individuals may contribute to a more equitable health care	Medicare, Interpretative Phenomenolo gical Analysis, Mental Health, Advocacy, Federal Legislation

							system for Medicare beneficiaries.	
40.	Battams & Henderson (2012)	To examine the current and potential impact of the United Nations Convention on the Rights of Persons with Disabilities (CRPD) on Australian and South Australian legislation and policy.	Qualitative interviews.	Ten interviews with professionals from law, psychiatry, policy and service user advocacy backgrounds working at state, national (Australian) and international levels and 3 with people with lived experience of disability.	Australia, South Australia, and international.	The interviews addressed three key themes: 1. the rights of people with psychiatric and other disabilities; 2. perceptions of the rollout of the United Nations Convention on the Rights of Persons with Disabilities (CRPD); 3. and the impact on current policy and practice of the 'right to health'. The authors find medical discourse of disability tied to the mental health sector, the lack of linkages between international, national and state legislation and policy processes, a lack of political commitment to the CRPD, and separation between the mental health and disability sectors are some of the barriers to the successful roll out of the CRPD.	The research highlights the rollout of the United Nations Convention on Persons with Disability and the achievement of the 'right to health' for people with psychiatric disability. Stronger links between human rights, disability and mental health sectors and political advocacy and engagement with CRPD official and shadow reporting processes would provide some hope for progress in implementing and giving reality to the 'right to health'.	Human Rights, Mental Illness, People with Disabilities, Psychiatric Disability.
41.	Agrusti et al. (2020)	To provide an overview of the community Needs Assessment (CNA) methodology	2018 social services mixed methods communit	Eight socio-economically disadvantaged	United States.	The authors find several barriers and challenges to meeting the local needs with residents reporting an inability to access necessary mental health services. The	This case example based on the methodology used sheds light on various policy and program factors. In addition, it serves as an exemplary	Community Needs Assessment, Mental Health Needs, Local

		applied, and the methods that generated valuable insights for the Hillsborough County Social Services Department (Social Services) and their community action board.	y needs assessmen t of eight socio-economica lly disadvanta ged neighbour hoods.	neighbourh oods.		community listening sessions provided authors with themes on how behavioural health disorders have made life difficult for lower-income residents.	model where academia and government agency collaboration can extract new insights that support government policy innovation to meet local mental health service needs	Policies, And Programs.
42.	Österman et al. (2014)	To present the changes in exposure to various types of physical punishment (including by parents) towards respondents born between 1931 and 1996, investigating the 28 years after the law prohibiting all kinds of physical punishment towards children.	Quantitati ve questionna ire.	Representat ive sample of citizens in Western Finland aged between 15 and 80 years (2,632 female and 1,977 male respondents).	Finland.	The results indicated a significant drop in reports of being slapped and beaten with an object among respondents who were born after the law was adopted. Associations between the decline in physical punishment with a similar decline in the number of murdered children was also seen. Exposure to higher or more frequent amounts of physical punishment than average was associated with significantly higher score on alcohol abuse, depression, mental health problems, schizotypal personality, divorce	The authors suggest that while a significant improvement in the safety of children in Finland has been reached, much still remains to be done, proposing that a decline in physical punishment of children does not occur automatically but that the implementation of a law is necessary as the decreasing amount of victimization from physical punishment after the law is clearly reflected in the steadily increasing percentages of respondents who had never been	Physical Punishment, Legislation, Depression, Mental Health Problems, Schizotypal Personality, Alcohol Abuse, Divorce, Suicide, Filicide.

						later in life and suicide attempts in the past 12 months.	victimized during childhood. In order to diminish violence against children and ensure their human rights, a coordinated approach is essential, with information campaigns before, after, and the implementation of the law.	
43.	Steel et al. (2011)	To report the analysis of two-year follow-up data, examining specifically for differences in the trajectory of psycho-logical symptoms and key indices of social adaptation amongst permanent protection visa holders (PPVs) and temporary protection visa holders (TPVs).	Longitudinal survey over 2 years.	104 consecutive refugees from Afghanistan and Iran speaking the Farsi and Dari dialects attending a state-wide early intervention program in New South Wales.	Australia.	The results indicated that TPVs had higher baseline scores than PPVs on the HTQPTSD scale, the HSCL scales, and the GHQ. Models adjusting for baseline symptom scores indicated an increase in anxiety, depression and overall distress for TPVs whereas PPVs showed improvement over time. At follow-up, TPVs maintained high PTSD levels, increased worry, and showed no improvement in English language skills, exhibited prolonged social withdrawal and persistent distress in handling post-migration challenges; compared to PPVs exhibiting substantial language improvements and becoming more socially engaged, also	The authors suggest a pattern of growing mental distress, ongoing resettlement difficulties, social isolation, and difficulty in the acculturation process amongst refugees subject to highly restrictive (including long term detention) immigration policies.	Australia, Refugees, Immigration Detention, Policies of Deterrence, Psychosocial Outcomes, Mental Health.

						reporting fewer problems in meeting these resettlement challenges.		
44.	Pinheiro & Sousa (2020)	To describe the legislative construction aimed at protecting the rights of children and adolescents and, to present some of the results obtained from the analysis of the speeches of the subjects interviewed, in order to highlight the perceived distance between the legal provisions and the practices of the courts.	Qualitative interviews.	Eight interviews with legal professionals working with lawsuits involving children.	Brazil.	The analysis of semi-structured interviews with legal professionals who work with lawsuits involving children, indicated an objectification of the children and the distance between the positivation and the effective fulfilment of the children's rights despite the legislation of the integral protection of the child in force for almost 30 years and still so unknown in the everyday life of the courts.	The authors suggest that it is imperative to understand and comply with, now and in the future, the status of subject of rights that children have especially children who have been judged, in order to see the perpetration of injustices to promote the dignified treatment of them. In addition it is necessary to consider the enormous contingent of lawsuits that discuss the lives of children who are processed in the Family Courts and Children's Courts in the country and it is relevant to recognize the urgent need to materialize, in the practical-theoretical, professional and institutional plans, the legislation of the integral protection of the child, which enshrines him as a subject of rights, and no longer an object.	Child, Justice System, Social Justice, Civil Rights, Social Psychology.

45.	Fisher & de Mello (2011)	To discuss how the findings of a recent systematic review of mental health problems in adolescents in resource-constrained settings might be applied using the 4-S Framework.	Systematic review.	Adolescents in resource-constrained settings.	International.	The findings suggest that in the absence of evidence about effective interventions in resource-constrained country settings, expert opinions indicate a broad public policy response which addresses direct strategies for prevention, early intervention and treatment; health service and health workforce requirements; social inclusion of marginalised groups of adolescents; and specific education is required.	The authors find that the predominant endorsed action is not that dedicated mental health services for adolescents are required, however that mental health care should be integrated using cross-sectoral strategies into the communities in which adolescents live, the institutions they attend and the organisations in which they participate.	WHO | 4S Framework | Mental Health Problems | Policies | National Strategies | Mental Health Services | Adolescents | Resource-Constrained Countries | Cross-Sectoral Strategies | Adolescent Psychology | Mental Disorders | Mental Health | Health Care Policy | International Organizations | Strategies | Adolescent | Health Resource

46.	Lenta & Zaldúa (2020)	To present an analysis of multiple-case studies comprising typical cases of rights violations from a community psychology approach.	Qualitative, including life stories, focus groups and naturalistic observation.	Children and adolescents. Participants were 16 boys and girls, five men and 11 women, intentionally selected from their attendance at two social programs for the restitution of the rights of children and adolescents.	Argentina.	The analysis of the cases presented led the authors to question the effectiveness of social policies aimed at restoring people's rights as the participants' life trajectories revealed micro- and macro-political obstacles to accessing rights, which is a situation that supports the social reproduction of inequalities and has de-subjectivizing effects.	The authors suggest that situations that guarantee identity grounding and rights enforceability may promote individual and collective subjectivizing processes in the actors involved and pose new challenges in the construction of one's life project.	Community Social Psychology, Childhood, Life Trajectories, Social Policies.
47.	Newbigging et al. (2015)	To report findings from a national review of Independent Mental Health Advocate (IMHA) provision in England.	Qualitative, including focus groups and interviews.	289 participants – 75 focus group participants and 214 individuals interviewed – including 90 people	England.	The findings indicate that the experience of compulsion can be profoundly disempowering, which confirms the need for IMHA, as access is highly variable and more problematic for people with specific needs relating to ethnicity, age and disability. In addition, satisfaction was reported more	The authors find that IMHA services have the potential to significantly shift the dynamic so that service users have more of a voice in their care and treatment. However, a shift from a narrow conception of statutory advocacy as safeguarding rights to one	Empowerment, Evaluation Research, Mental Health Act, Mental Health Advocacy, Policy

				eligible for Independent Mental Health Advocate services, alongside advocates, hospital and community-based mental health professionals, and commissioners.		frequently in terms of positive experiences of the process of advocacy, rather than tangible impacts on care and treatment under the Mental Health Act. The uptake of IMHA services was influenced by available resources, attitude and understanding of mental health professionals, as well as the organisation of IMHA provision.	emphasising self-determination and participation in decisions about care and treatment is needed; in addition, access could be improved through a system of opt-out as opposed to opt-in.	Research, Users Views, Rights
48.	Moss & Vollhardt (2016)	To investigate the interview responses of 56 Rwandans to single subordinate ethnicity recategorization policies.	Qualitative interviews.	56 Rwandans.	Rwanda.	The responses indicate that despite strong societal pressures, a variety of positions were apparent, which shows how people actively construe categories, and the complexity of single recategorization in real-world settings.	The authors suggest several novel insights and policy implications, including the need for context-specific approaches, utilitarian reasons for single recategorization, the role of malleability of identities in promoting social recategorization, and the potential use of recategorization models as transitional measures in post conflict societies.	Government Policy Making | Genocide | Social Psychology | Ethnic Groups | Developing Countries | Racial and Ethnic Groups | Syringes

83.	Kenny et al. (2017)	To review the U.S. laws on child maltreatment reporting as they pertain to psychologists and to provide direction to psychologists in interpreting state laws as well as practical and clinical guidance when confronted with making a report of child abuse.	Conceptual paper (review of US laws on child maltreatment reporting).	Psychologists.	United States.	The reviewed laws and statutes in all 50 U.S. states and the District of Columbia (DC) were analysed for themes in the following content as: (a) mandated reporters and reporting procedures (e.g., threshold and time frame for reporting, content and method of reporting); (b)types of abuse defined (physical, sexual, neglect, and emotional abuse); (c) immunity for reporters; and (d) failure to report and false reporting. The findings suggest that while these statutes may seem straightforward, numerous grey areas in reporting cases of abuse exist, such as the decision to inform involved caregivers and the issues surrounding encouraging family members to make a CPS report themselves.	The authors stress that psychologists must be aware of their mandatory obligation to report and reduce the impact of childhood maltreatment by keeping informed of the state laws regarding child maltreatment and being aware of their local child protection agency. This article provides information about the legal implications and protections put in place for psychologists who report in good faith with suggestions for training programs that should include child abuse reporting information as part of their curriculum to prepare trainees to exercise their legal and ethical responsibility when confronted with the unavoidable reality of working with victims of child abuse.	Mandated Reporters, Psychologists, Child Maltreatment, U.S. Laws, Ethics

2) Conceptual Articles

Article No:	Authors:	Aims:	Type of Article:	Population of Interest:	Geographical Location:	Main Conclusions:	Key Words/ Topics:
1.	Cronin et al. (2017)	To outline similarities and differences with regard to mental health legislation between five jurisdictions: Republic of Ireland, England and Wales, Scotland, Ontario (Canada), and Victoria (Australia).	Narrative review.	People with mental health problems who are involuntarily treated and detained.	Republic of Ireland, England and Wales, Scotland, Ontario (Canada), and Victoria (Australia).	Across the jurisdictions, broadly similar procedures for admission, detention and treatment of involuntary patients are implemented, signifying compliance with human rights principles. However, there are differences with regards to the criteria in defining a 'mental disorder', automatic review hearings subsequent to a patient being involuntarily admitted, and supported decision-making under mental health legislation.	Consent To Treatment, Mental Health Act 2001, Mental Health Legislation, Mental Health Tribunals.
27.	Petersen et al. (2016)	To identify 'best practice' and 'good practice' interventions that can feasibly be delivered at population (PL)- and community (CL)-levels in low- and middle-income countries (LMICs), to aid the identification of resource efficiencies and allocation in LMICs.	Narrative review.	People with mental, neurological and substance use disorders.	Low- and middle-income countries.	The authors identify 'best practices' at the PL as: laws, and regulations; and at the CL: socio-emotional learning and parenting programmes; in addition to 'good practices' including Child protection laws, improved control of neurocysticercosis and public campaigns. This highlights the need for training non-specialist community members at a neighbourhood level to assist with community-based support and rehabilitation of people with mental disorders. Interventions provided at the population- and community-levels have an important role to play in promoting mental health, preventing the onset, and protecting those with MNS disorders.	Mental Health, Community, Population-Level, Low-And Middle-Income Countries

39.	Evans & Russell-Mayhew (2020)	To discuss the future directions for social justice and advocacy in counselling psychology in relation to systemic change.	Conceptual paper	Working group entitled "The Responsibility of Canadian Counselling Psychology to Reach Systems, Organizations, and Policy-Makers", including students, researchers, clinicians, and professors (N=12).	Canada.	The discussions at the 2018 Canadian Counselling Psychology Conference generated three main themes: identifying needs, using the unique training, and infiltrating and navigating the system; by echoing the definition of social justice in counselling psychology as "both a goal and a process for counsellors who believe in developing an increasingly socially just world". The authors hoped to create a compelling argument to move toward implementing more formal guidance in counselling psychology programming and professional development to support the process of advocacy, as opposed to social justice remaining an aspirational goal that is attempted by individuals alone.	Counselling Psychology | Needs | Counsellor Role | Systemic Change | Counselling Psychologists | Organizations | Social Justice | Advocacy | Systems | Canada | Organizational Change Health Service Needs | Needs Assessment | Policy Making | Change Strategies | Self Psychology
49.	Kitafuna (2022)	To provide a critical overview of mental health-related beliefs, services and systems in Uganda, alongside recent activist and legal challenges. The overview aims to examine the deeper	Conceptual paper.	Ugandans with lived experience of mental health problems, including survivors and those still in treatment or care.	Uganda.	While Ugandans with mental health problems experience significant stigma, social exclusion and punitive treatment, incremental improvements and revolutionary advancements have been evident in the last 2-3 decades as a result of momentum by activists with psychosocial disabilities, in addition to the UNCRPD, international disability alliances, and international law.	Human Rights, Global Mental Health, Service User Activism.

		context to inform current reform efforts.					
50.	Wong et al. (2014)	Critically explores mental health policies with regards to legislation, financing, model of care and delivery, in addition to the training of mental health professionals in China.	Theoretical (Review of policies).	People with mental health problems.	China.	The review raises numerous policy-related questions regarding the lack of community-based psychiatric services, poor standard of education and an insufficient number of trained mental health professionals, and inadequate protection of the rights of individuals with mental health problems. Underpinning numerous concerns, such as the need to strengthen mental health services in rural areas, there is a need for different levels of government, particularly the central government, to commit to improving mental health services for people with mental health problems in China.	Mental Health, Policies, China, Critical Review, Psychiatric Services, Mental Health Legislation.
51.	Cosgrove et al. (2020)	To examine the Lancet Commission's report and to argue that a focus on societal determinants, rather than more narrowly defined 'social' evident in the Report, and political economy, could provide new possibilities for global mental health beyond more narrow individualized medical interventions.	Conceptual paper (critical review).	Mental health activists and service-users.	Global.	While some attention was afforded to social determinants of health in the Lancet Commission Report, it adopts an intra-individual focus and privileges a (Western) medical and disease model. A paradigm shift is needed. Instead of conceptualising poverty, violence, or gender inequity as predictive variables or risk factors for mental disorders, we need to view the crisis in mental health as a crisis of obstacles. A 'global burden of obstacles' approach (one that addresses structural issues) aligns with a politically informed societal determinants of health framework and will move global mental health in a more emancipatory direction.	Global Mental Health, Lancet Commission, Social Determinants of Health.

52.	Brodsky et al. (2013)	To examine the question of whether psychologists should have involvement in death penalty cases.	Conceptual paper.	Defendants facing capital trials and punishment.	United States.	Unequivocally banning psychologists from participating in the capital legal process is not justified at present. Until a more universal perspective on capital punishment is reached, a nuanced and psychologically responsible approach may be a better way to protect the rights and dignity of defendants in capital trials.	Death Penalty Cases, Psychologists' Involvement, Capital Punishment, Professional Ethics.
53.	Thomas (2013)	To conduct a review of thinking and research about inclusive education policy.	Theoretical (review).	Disadvantaged school students.	Not specified.	There are two psychologies of learning with which inclusive education needs to engage. First, there are new models of learning that emphasise the significance of community, with an emphasis on meaning, narrative, apprenticeship (i.e., the context and culture for learning). Second, there is a new 'psychology of difference' based on gradient effects and the impact of these on identity. Inclusive education rests on its capacity to retreat from histories of identify-assess-diagnose-help and to explore the ways in which schools enable community and encourage students' belief in themselves as members of a community.	Inclusive Education, Equity, Review, Inclusion.
54.	Mitchell & MacLeod (2014)	To explore the key role that researchers and community health practitioners can play in protecting against the misrepresentation of community voices in neoliberal policy environments. The	Conceptual paper.	Aboriginal populations.	Canada.	An overview of Canadian Aboriginal social policy history indicates that public policy can be a crucial and destructive political tool that significantly affects community mental health.<br>Recommendations are made to increase the commitment of the community mental health profession to participation in the monitoring and advancement of social	Social Policy, Neoliberalism, Social Transformation, Aboriginal.

		authors examine social policy as a crucial contributor and mediator to the mental health and well-being of Aboriginal people.				policy processes that promote legitimate community consultation and community wellness. The authors promote building relationships with Aboriginal communities to advance social policies underpinned by an understanding of colonial history and the cultural world views of specific Aboriginal populations.	
55.	Uribe Aramburo (2011)<br><br>Could not find translated document.	To examine the phenomenon of child sexual abuse and identify the administration of justice in Colombia with regards to clinical and forensic psychology.	Conceptual paper.	Children and adolescents.	Colombia.	The authors highlight the knowledge gap in clinical psychology in the legal context of child sexual abuse cases in Colombia. The authors also stress the need for addressing these challenges, including legal contradictions and lack of resources, to improve the administration of justice. The proposed solutions include interdisciplinary dialogue and training for legal professionals; identifying and categorising various problems within legal proceedings and advocating for psychologists and jurists to engage in further research and procedural refinement to address the complexities of child sexual abuse cases in Colombia.	Child Sexual Abuse, Justice Administration System, Legislation, Jurisprudence, Child Development.
56.	Gilfoyle & Dvoskin (2017)	To provide an overview of APA’s Amicus Curiae Program.	Conceptual paper.	Psychologists; judicial decision-makers.	United States.	The authors find that psychological science will be relevant to future legal issues, whereby APA's amicus curiae programme, which translates psychological research findings to the courts on key public law issues, has been a crucial mechanism in creating, communicating, and applying psychological knowledge to benefit society	Law And Psychology, U.S. Supreme Court, Amicus Curiae, Briefs

						by facilitating fair and informed decisions by the courts.	
57.	Magor-Blatch (2011)	To provide a framework, beyond the 'Zero Tolerance' philosophy, to promote social justice and healthy adolescent development.	Conceptual paper.	Children and adolescents.	Australia and United States.	The 'Zero Tolerance' philosophy promises more than it can deliver, and its impact is frequently misunderstood by its supporters. Its use in schools has resulted in schools becoming reactive rather than proactive with regards to antisocial and problem behaviours. Understanding adolescent development and working more proactively within a harm minimisation and primary prevention model will provide better opportunities for at-risk children and young people.	Zero Tolerance, Adolescent, Drug Abuse, Primary Prevention, Restorative Justice.
58.	Valentim (2013)	To provide a commentary on Brazilian social psychology in the international context.	Conceptual paper (commentary).	Psychologists.	Brazil.	With regards to science policy and education policy, changes in this area have included the gradual devaluation of university pedagogy in favour of the research component, both in the assessment of individuals and institutions. This has occurred alongside a decrease in the use of other languages in favour of English as the language of international scientific communication and the preference for publishing articles rather than books.	Brazilian Social Psychology, Intergroup Relations, Colonialism, Science Policy, University Pedagogy.
59.	Forsman et al. (2015)	To present selected research initiatives, 2007–2014 that contribute to efforts of realizing the mental health priorities of the Nordic Council of	Conceptual paper.	Nordic countries.	Denmark, Finland, and Sweden.	The public mental health research conducted at the NHV highlighted the complexity of mental health and emphasized that the broad determinants of mental health need to be increasingly addressed in both public health research and practice. Efforts to realize these priorities included commissioning the	Public Mental Health, Nordic Countries, Policy Analysis, Register Studies, Systematic Mapping

		Ministers in 2010 signalling a mutual Nordic exchange of knowledge in the following thematic areas: child and adolescent mental health; working life and mental health; mental health in older people; strengthening the role of primary care in mental health service provision; stronger involvement of users and carers; and reduction of use of coercion in psychiatric care.				Nordic Research Academy for Mental Health to develop, organize and follow-up projects on public mental health. The research initiatives included: mental health policy analysis, register-based research and research focused on the users' perspective in a Nordic context, as well as EU-level research policy analysis. By exchanging knowledge and best practice, the collaboration between the Nordic countries contributes to the welfare of the region. The expertise and traditions developed at the NHV are of significant importance in this work.	
60.	Christie & Montiel (2013)	American Psychologists contributions to policies that promote peace, social justice, and human well-being are discussed and the growth, scope, and content of peace psychology are reviewed in this article.	Conceptual paper.	Psychologists.	United States.	The contributions of American psychologists to war have been substantial and responsive to changes in U.S. national security threats and interests for nearly 100 years. These contributions are identified and discussed for four periods of armed conflict: World Wars I and II, the Cold War, and the Global War on Terror. While about 50 years ago some psychologists in the United States and around the world broke with the tradition of supporting war and began focusing their scholarship and activism on the prevention of war and promotion of peace leading to	Peace Psychology, Negative Peace, Positive Peace, Cycle of Violence, Conflict Resolution

						today's peace psychology as a vibrant area of psychology, with theory and practice aimed at understanding, preventing, and mitigating both episodes of organized violence and the pernicious world-wide problem of structural violence.	
61.	Rami et al. (2022)	To provide an overview of the impact of COVID-19 on healthcare disparities and particularly its impact on marginalized com munities.	Conceptual paper.	Psychologists; and marginalised communities.	International.	The authors emphasise the importance of addressing healthcare disparities globally, advocating for mental health, and promoting ethical models for a more inclusive and equitable future; as the article highlights the widespread impact of COVID-19, with consequences on mental health, healthcare disparities, and socioeconomic inequalities by exploring ethical considerations in public health responses and the challenges faced by vulnerable populations. The article reveals the pre-existing issues and calls for integrated solutions, including innovative approaches like telehealth.	International Psychology, Healthcare Disparities, Ethics, Mental Health, Covid-19.
62.	Moreno et al. (2020)	To highlight the historical development of LGBTQ psychology i n the context of different realities for LGBTQ people in five different countries.	Conceptual paper.	LQBTQ.	Russia, Greece, Colombia, South Africa and the province of Quebec, Canada.	While various forms of homonegativity and transphobia still coexist even in countries that have established protections and rights recognition for LGBTQ people, the cross-cultural narratives show that the recognition of lesbian and gay rights comes before the recognition of the rights of bi, trans, queer and questioning people. The authors provide 15 strategies to help psychological organizations and psychologists around the world promote the development of LGBTQ	LQBTQ, Psychology, Russia, Greece, Colombia, South Africa, Canada, Quebec.

						psychology as human and mental health sciences have played an important role in the blossoming of LGBTQ psychology.	
63.	Lea (2021)	To review psychological studies of real-life use of credit, debt, and over indebtedness, with the aim of making policy recom mendations that could reduce the damage done by debt to both individuals and society.	Conceptual paper.	Socioeconomica lly disadvantaged individuals; psychologists.	Not specified.	The authors suggest that even if the recommendations formulated in this article were adopted overnight, the problems of debt in society would not go away as credit enhances consumer choice and is a necessary function in a modern economy. Appropriate policies proposed here, could reduce both: 1. Reduce poverty, 2. Maintain and intensify regulation of high-cost lenders, 3. Avoid unnecessarily labelling financial support as a loan. 4. Educate children in financial calculations, 5. Give extra protection to those vulnerable to debt when offering credit, 6. Enhance people’s awareness of their credit position, 7. Advise debtors to approach creditors only with appropriate support.	Psychological C auses | Psychological Consequences | Policy Implicat ions | Debt | Economic Inequ ality | Social Insecurity | Financial Strain | Policy Making
64.	Borg (2010)	To explore some of the dynamics of our culture’s approach to dealing with difference, especially when manifested in disenfranchised individuals.	Conceptual paper.	Disenfranchised individuals; people with mental disabilities.	United States.	The author suggests that as long as we live in a culture that develops and implements repressive social policies, we are all culpable for the oppressive behaviours that such policies support. The ACT program and offshoot projects such as the Collaborative Mental Health Initiative exemplify how it is possible to address the needs of groups too frequently overlooked in social justice discourses. When we look beyond the	Ableism, Assertive Community Treatment, Civil Rights, Disability, Kendra’s Law

						language of individual pathology to the more threatening issue of how we treat marginalized populations, we expose frighteningly the degree to which many people in this society still lack the security and resources they need for safety, empowerment and well-being.	
65.	Wolf et al. (2013)	To present a model and theory of change based in a select, but diverse, array of psychological theories (self-efficacy theory, self-determination theory, and bioecological systems theory) for how conditional cash transfers (CCTs) may operate to affect families and child development.	Conceptual paper.	Families and children.	International.	This article touches on relevant evidence supported by select psychological theories to help shed light on the paths through which CCTs may affect some processes of child development. While there is evidence for the positive effects of CCTs on certain outcomes, there are many areas of human development and Behavior potentially affected by CCTs that still need to be conceptualized, measured, and evaluated. The authors suggest possible psychological mediators and outcomes that may be critical in evaluating the success of current and future CCT programs, as using a guiding framework in research and design of social policy is critical for improving policy efforts.	Conditional Cash Transfers, Psychological Theory, Child Development
66.	Fine (2013)	This article challenges the widening epistemological gap between those who suffer from inequality and those who conduct	Conceptual paper.	Incarcerated women.	United States.	The authors are inviting readers to explore how critical participation by a collaborative team of university and prisoner researchers has facilitated theoretical and methodological complexity, enhanced contextual and construct validity, strengthened commitments to ethics and	Participatory Action Research, College in Prison, Activist Research

		social policy research on inequality in response to Martin Luther King Jr.'s 1968 address at the American Psychological Association calling for a psychology that would educate Whites about racial injustice.				action, and fuelled the political sustainability and generalizability of the findings over time and space in this 20-year memoir on the echoes of a single piece of participatory policy research, Changing Minds: The Impact of College in a Maximum-Security Prison (Fine et al., 2001).	
67.	Carew et al. (2010)	To question how psychologists can be more involved in contributing towards encouraging people to believe that they can contribute to society, and how can psychologists help people to recognize they have the assets and talents to do so?	Conceptual paper.	Psychologists.	UK.	The authors conclude that health and well-being are central to improving working life and reducing the risk of social exclusion through unemployment and suggest that psychologists can be more involved with a supportive approach for individuals and employers towards helping people view work as a real possibility, particularly those who experience mental health problems. Psychologists have a significant contribution to make here to help bridge the gap between inclusion and exclusion as this is an exciting time for policy with this emphasis on enabling as many people as possible to progress into and remain in work for as long as they would wish by accepting early intervention as the key.	UK Government | Social Exclusion | Social Inclusion | Employment | Policy | Psychologists | Employment Status | Government Policy Making | Social Acceptance | Public Policy |
68.	Chaplin & Taggart (2012)	To bring readers up to date with policy developm ents in England and	Conceptual paper (policy and law update).	People with intellectual disability.	Northern Ireland and England.	The authors suggest that despite shared visions of inclusion and equality in mental health care, there are major differences in how both countries approach these issues.	Equality, Inclusion, Intellectual Disability,

		Northern Ireland in the last two years since the journal first reported on these issues of relating to mental health and intellectual disability.				While policy initiatives in both countries have been encouraging, they need to be implemented so issues such as access to healthcare and geographical disparity are addressed by local providers. In conclusion there is a desire for change, the reality is that the policies and legislation introduced to address issues such as accessing mental health care and service standards have still to make a significant impact to people's daily lives.	Legislation, Mental Health Promotion, Policy, Learning Disability, Strategy, Mental Health Services, United Kingdom
69.	DeBoer et al. (2022)	To outline the challenges as well as the importance of incorporating culture into a conceptualization of universal mental health in schools.	Conceptual paper.	Students from culturally and linguistically diverse backgrounds, who are more likely to experience barriers to mental health services.	Not specified.	The authors suggest that the current methods of addressing mental health at school are reactive and insufficient, failing to address the needs of all students and propose that without an explicit focus on dismantling systemic racism and inequity within schools, our society will continue to underserve those already disenfranchised. Challenges and the crucial role of incorporating culture into universal mental health are highlighted with proposed solutions spanning state-, district-, and school-level strategies, emphasizing equity-focused mental health practices within a multitiered support system. Recommendations include stakeholder support for structural changes, alongside school-level actions like fostering cultural humility, creating a culturally responsive climate, and conducting culturally sensitive assessments.	Equity, Multitiered System of Support, Research-To-Practice, School Mental Health, Tier 1 Services

70.	Zayas & Bradlee (2014)	To offer a summary of how U.S. citizen-children come into contact with the immigration enforcement system by exploring the impact of detention and deportation on the health, mental health, and developmental trajectories of citizen-children and arguing for reforms in policy and practice that will adhere to the highest standards of child welfare practice.	Conceptual paper.	Children of immigrants.	United States.	The authors offer a summary of how U.S. citizen-children come into contact with the immigration enforcement system by discussing the de facto classes of children: exiles and orphans, as immigration policies frequently creates such; and suggest that by integrating these children into the immigration discourse, practitioners and policy-makers will be better able to understand the effects of immigration enforcement, reduce harm to children, and provide for the protection of their rights.	Child Mental Health, Citizen-Children, Deportation, Immigration Enforcement, Mixed-Status Families
71.	O'Donnell (2012)	To identify the core Global mental health (GMH) resources to further orient those with various levels of experience in the mental health, psychology, and health fields in solidarity with the emerging GMH movement, which focuses on improving	Conceptual paper.	Socioeconomically disadvantaged populations.	International.	The authors explore six “resource areas” with an emphasis on the last two decades: organizations, publications, conferences, training, human rights, and the humanitarian sector and present the article as a “resource primer” to promote and guide the integration of mental health into our personal and global agendas for human wellbeing. As the GMH domain is full of an ever-expanding array of relevant resources, colleagues are encouraged to: use this primer in training settings, build on it with additional materials from international sources, and expand it to include additional resource areas. Four	Global Mental Health, Health Disparities, Humanitarian Psychology, International psychology, Mental Health Resources, Wellbeing

		wellbeing and mental health equity.				suggestions to support people from a variety of backgrounds and different levels of international experience include: regular GMH updates; perseverance, embracing lifestyles that reflect commitments to equality, justice, and wellbeing for all; despite challenges to celebrate the GMH progress as the "social progress and better standards of life in larger freedom".	
72.	Fisher (2013)	To raise questions for ethical analysis of psychologists' participation in another kind of controversial legal proceeding; death penalty cases.	Conceptual paper.	Psychologists.	United States.	The author, explores the ethical dilemmas surrounding psychologists' involvement in death penalty cases with focus on the flaws in the death penalty process, including the potential for wrongful convictions, racial and socioeconomic inequities, and the fallibility of psychological assessments. In addition, questions are raised whether forensic psychologists' participation in capital cases, given these issues, can be justified without violating human rights suggesting that the moral stance against psychologists' involvement in military interrogations justifying human rights violations may extend to the death penalty, an equally inequitable legal process with lethal consequences for defendants.	Human Rights | Psychol ogist Involveme nt | Death Penalty | Legal Processes | Capital Punishment | Professional Ethics | Psychologists | Humanities | Humanism | Humans
73.	Kinscherff & Grisso (2013)	To discuss issues arising from the amendment of APA ethical Standard 1.02, which clarified that psychologists may not	Conceptual paper.	Psychologists; Juveniles.	United States.	The authors conclude that without clear direction from APA as to how psychologists are to identify the sources of "human rights" principles and law applicable under Standard 1.02, it will be very difficult except in the most extreme and obvious situations	Human Rights Violation s | Human Rights Laws | Juvenile Capital Cases |

		rely upon the standard to justify professional participation in even lawful activities if doing so would 'justify or defend violating human rights'.				to discern what circumstances or professional practices constitute or collude with violations of human rights. Ethical questions are raised whether a psychologist would be subject to sanction under Standard 1.02 for professional conduct that is lawful in the United States but would violate international human rights law or standards not embraced under American law or what would constitute a violation of human rights sufficiently egregious or systematic that a psychologist must should disengage or continue participation to protect individuals, act as an internal whistleblower, or remain a witness in the hope of intervention to stop ongoing human rights violations.	Psychologists | Adjudication | Human Rights | Professional Ethics | Humans | Humanities | Humanism
74.	Carr & MacLachlan (2014)	To present a manifesto for tackling global inequalities at work by questioning how psychology, in close conjunction with other social sciences is currently affecting policy and practice in international development; and in what ways can psychology make a stronger future contribution to interdisciplinary	Conceptual paper.	Not specified.	Not specified.	The authors argue that the scope and interdisciplinary nature of the field of psychology confers an obligation to influence international development policy.	Humanitarian Work Psychology | Environment | International Development Policy | Social Sciences | Poverty | Foreign Policy Making | Prosocial Behavior | Psychology | Self Psychology

		approaches to poverty reduction.					
75.	Ward et al. (2018)	To introduce normative multiculturalism, propose an integrative framework, examining its impact in terms of intergroup relations and subjective well-being; while focusing on exploring how immigrants and the receiving community may differentially experience multiculturalism at the societal level by discussing strategies for maximizing the benefits and minimizing the risks of multiculturalism.	Conceptual paper.	Heterogeneous cultural groups in multicultural societies.	Not specified.	The authors contend that cultural diversity is a fundamental feature of multiculturalism, but that the ideological appreciation of this diversity and its accommodation multicultural policies and practices that ensure cultural maintenance and inclusive, equitable participation are equally important. A conceptual framework that integrates the contextual antecedents and the psychosocial outcomes of multiculturalism and have proposed directions for future research is provided in addition to suggestions for approaches to maximize the benefits and minimize the risks of cultural diversity for both majorities and minorities are offered.	Multiculturalism | Immigration | Intergroup Relations | Subjective Well-Being | Social Justice | Diversity | Ideology | Policy | Psycho logical Research Experimentation | Intergroup Dynamics | Policy Making | Psychology | E migrants and Immigrants
76.	Torres et al. (2018)	To review current immigration policies for arriving Mexican and Central American immigrants and links to mental health among documented and undocumented	Conceptual paper.	Latinos.	United States.	Authors discuss positive policies and resources that may mitigate the damaging impact of immigration-related stress; highlighting several policies and laws that have significant negative mental health-related implications for immigrant families and children, including limited access to public benefits and health care, and the devastating effects of deportation; and	Latino/A Immigrants, Immigration Policy, Immigration-Related Stress, Trauma and Mental Health,

		immigrant families and youth.				bringing to attention a greater need for clinicians and researchers to not only become aware of the issues impacting Latino/families, but also to advocate for policies that reduce risk for additional traumas and distress.	Resilience and Social Justice
77.	Bendat (2014)	To expound on the Affordable Care Act (ACA) and parity laws with an eye toward patient advocacy and empowerment of mental health professionals given the profound, cur-rent need for treatment and continued insurer impediments to meaningful care: recommendations included.	Conceptual paper.	Mental health and substance abuse treatment service users.	United States.	The authors find that while the ACA has mandated a reduction in healthcare costs, including administrative fees, the ACA does not set uniform reimbursement benchmarks for any size plan, whether fully insured or self-funded and that without Congress and the states acting swiftly to establish minimum reimbursement benchmarks, private insurance will be nothing more than an extension of Medicaid—with meaningful care effectively out of reach for premium-paying patients lured into the ACA exchanges by the promise of access, affordability, and quality. Even with the promise of mental health parity and "essential health benefits," access to care is meaningless in the absence of reasonable re-imbursement benchmarks as many insurers have set the reimbursement bar so low that some leading facilities and providers have refused their compensation terms.	Mental Health Parity | Substance Abuse | Patient Protection Reforms | Legislative Remediation | Legislative Processes | Health Care Policy | Health Care Reform | Drug Abuse | Parity
78.	Melton (2010)	To analyse the United Nations Convention on the Rights of the Child (CRC), focusing on its role in making	Conceptual paper.	Children.	United States.	The authors discuss the potential impact of CRC ratification on addressing current issues by emphasizing the transformative potential of the United Nations Convention on the Rights of the Child (CRC) in shaping	Child Mental Health | Convention on The Rights of The Child |

		government action to support relationships crucial for children's development and protection by delving into CRC provisions concerning the right to personality, mental health, and challenges in establishing a universally accessible child mental health system; and considering the potential impact of CRC ratification on scrutinizing and addressing current issues in child mental health.				the status of children globally by underscores the CRC as a blueprint for legal structures promoting a climate where children are treated with respect, aligning with psychological research on perceived justice and advocating a dual-maximal approach, ensuring fairness while recognizing the need to enhance conventional human rights for children's meaningful participation in society. The CRC's obligations to support children's identities, growth, and protection, especially in mental health, are highlighted, along with challenges in building an effective child mental health system.	Child Welfare | Child Policy | Child | Child Advocacy | Global Health | Humans | International Cooperation | Mental Disorders | Mental Health | Psychology | Societies, Scientific | United Nations | Government Pol icy Making | Human Rights | Only Child
79.	Crocker et al. (2010)	To provide the readers with a brief introduction to the history of Canadian law regarding the administration of mentally disordered accused and the challenges in applying these laws in the large and diverse nation that is Canada by	Conceptual paper.	Individuals who are found not criminally responsible on account of mental disorder.	Canada.	Authors conclude that the, the number of mentally disordered accused under the jurisdiction of Review Boards has increased steadily since Criminal Code changes in 1992 in Canada similar to the United States, where a steady increase over a similar time period in the number of persons admitted to state mental health facilities after being found Not Guilty by Reason of Insanity. This article highlight concerns about the potential transformation of mental health systems into de facto forensic systems as the	Not Criminally Responsible Due to Mental Disorder | Mentally Ill Offenders | Mental Health Services | Forensic Mental Health Services | Social Services | Families |

		exploring discrepancies in the expertise of forensic assessments and thus in potential outcomes and trajectories of individuals found Not Criminally Responsible due to Mental Disorder using examples from Ontario, British Columbia (BC), and Quebec that together account for the large majority of NCRMD cases.				diversity in provincial systems, along with variations in mental health and criminal justice practices, may impact the consistency and quality of forensic assessments raising questions about the adequacy of treatment, potential prolonged detention, premature releases, and community safety risks for Not Criminally Responsible on Account of Mental Disorder (NCRMD) individuals.	Criminal Justice System | Service Access | Criminal Justice Treatment Barriers | Family | Forensic Psychology | Insanity Defence | Mental Disorders | Canada
80.	Mowat (2020)	To interrogates the relationship between poverty, attainment and children's mental health and wellbeing to inform public policy and practice in Scotland and beyond.	Conceptual paper.	Children.	Scotland.	Authors identify a key theme from the analysis of the literature as the nature, quality, and strength of social and support networks around schools, families, and children, which is contextualised within a discussion of the nature of childhood poverty and mental health in children in Scotland and the response of the Scottish Government and argue that focus on educational policy and schools alone will not in itself address the poverty-related attainment gap. They propose that holistic focus is required on public policy, informed by interdisciplinary research, in addition to focus on building a strong infrastructure of	Poverty, Attainment, Mental Health and Wellbeing, Policy, Social Support, Social Networks.

						support around schools, families and communities.	
81.	Scanlon & Adlam (2013)	To analyse the ethical implications of social policies implemented through the welfare state with the espoused objective of achieving social inclusion, drawing on ancient Greek philosophy contemporary psychosocial theorists.	Conceptual paper.	Not specified.	Not specified.	The authors argue that social policies establish a boundary between domains of inclusion and exclusion that perversely creates and maintains the very problem such policies are designed to solve; and provide 'rationalisations' for social exclusion which imply that such states can be explained –that they are ethical, and so legitimate by illustrating the argument with reference to a specific area of social policy-Improving Access to Psychological Therapies(IAPTA) derived from the analysis of how this policy frames aspects of social exclusion (un(der)employment?) as a 'psychological' problem.	Worklessness, Colonisation, Inclusion, Citizenship, Cosmopolitanism, Perversity, IAPTA
82.	Barrera Rojas et al. (2021)	To present a review of the conditions that exist to elevate mental health as a human right in the state of Quintana Roo in Mexico.	Conceptual paper.	Not specified.	Mexico.	Authors find that there is already a minimum floor in legal matters at the federal level, even at the state level, even though initiatives have been presented from the government itself and from non-governmental entities and suggest that it is difficult to think that in In the short term, there are sufficient political, public policy and infrastructure conditions to position mental health as a human right in Quintana Roo; leaving the door open for this work to be the apex of the discussion on the need and urgency to generate public policies, both in legal terms, as well as budgetary,	Mental Health, Human Rights, Public Policies

						administrative, operational, and physical infrastructure that allows for the short, medium, and long term.	
84.	Lange & Williams (2011)	To discuss the Clinical, ethical, and legal issues related to families with both parental mental health issues and child protection concerns; a common clinical situation, however complex, and pluralistic ethical issues.	Conceptual paper.	Families with both parental mental health issues and child protection concerns.	Australia.	The authors suggest that where statutory child protection concerns exist, both the Mental Health Act and the Children's Protection Act are needed to inform the clinical decision-making for individuals in the family. The ethical model of Cooperation Despite Disagreement reflects the pluralistic ethical views inherent when working with families where there are also concerns at the level of statutory child protection service involvement, encouraging: the family and the services into active communication in order to achieve consensus or agreed points of difference and the development of collaborative practice between the adult sector and the child sectors of health and social services, thus developing a closer.	Child(ren), Child Protection, Ethics, Law, Mental Illness, Parent.
85.	Aranda (2016)	To offers an overview of immigration law, the challenges of performing culturally competent assessments and consequences of failing to do so, and the plight of a particularly vulnerable group:	Conceptual paper.	Immigrants; psychologists.	United States.	The authors propose that Psychologists must be familiar with cultural differences and be aware of nuances stemming from age, gender, gender identity, race, ethnicity, culture, national origin, religion, sexual orientation, disability, language, and socioeconomic status; while employing culturally sensitive and valid methods in their assessments and be aware of their own limitations, as working in the field of immigration calls for the highest awareness of and sensitivity to diversity and cultural	Asylum, Undocumented Immigrants, Global Mental Health, Cultural Competence

		unaccompanied children.				competence requiring proper training about the populations they serve, the conditions in their countries, and specific problems they face.	
86.	Grey et al. (2013)	To examine the literature for causal factors for continued mental health inequalities facing Black and minority ethnic (BME) groups and potential solutions.	Conceptual paper.	Black and minority ethnic communities.	UK.	The authors stress that despite ethnic inequalities in mental health being a concern for decades in the UK, a significant gap exists between policies and methods of implementation as past policies have failed to narrow the gap and conclude that: 1. The inequalities are irresolvable. 2.The interpretation that variation equates to inequality is wrong. 3.Solutions need a longer time frame to have the desired impact. 4.The wrong solutions or combination of solutions is applied. Service providers are called to work closely with people from BME communities prior to service design delivery while addressing barriers in terms of accessing culturally appropriate services, including lack of cultural understanding, communication issues, and where and how to seek help.	Mental Health Inequalities | Minority Ethnic Populations | Government Pol icy Initiatives | Communities | Government Pol icy Making | Racial and Ethnic Groups | Initiative | Mental Health | Socioeconomic Factors
87.	Chhabra & Kapadia (2023)	To bring the discrimination faced by the gender minority population caused due to the unfavourable attitudes, and stigma persisting at the interpersonal and community level into	Conceptual paper.	LGBTQ+ community.	Not specified.	The authors conclude that while many countries have progressed in designing favourable policies, a widegap in identifying the challenges faced by them in accessing the existing mental health services still exists. The LGBTQ community as one of the major minority communities globally, further strive to be seen and heard which is not very well received by the society; as	Mental Health, LGBTQ, Trauma, Policies, Queer Community, Gender Minority.

		focus and to propose a way forward.				their major concerns include traumatic experiences, subsequent loss which may be misconceived or overlooked, experiencing persistent barriers for voicing their needs, with the struggle amplifying especially in the realm of accessing mental healthcare. Suggestions for tailoring the policies created in accordance with the needs and requirements are also proposed in this article.	
88.	Cowan et al. (2011)	To reopen a debate around the potential impact of narrow conceptualisations of inclusion, or participation, of service users in current mental health policy develop ment and implementation.	Conceptual paper.	Mental health service users.	UK.	The authors offer a fresh analysis that particularly points to the potential value of service user groups considering alternative forms of involvement, rather than those prescribed by ‘‘Third Way’’ or ‘‘Big Society’’ thinking. The conceptual analysis of the continuity of ‘‘New Labour’ thinking’’ findings show the potential for disempowerment and argue for alternative service user action, either contracting on ‘‘their own rules of engagement’’ or specifically taking up an oppositional stance to disempowering forms of involvement, while drawing attention to the influence of differing English and Scottish policy drivers which appear to offer potentially different forms of engagement.	Service User Involvement, Social Inclusion, Participation, Co-Option, Citizenship
89.	Das (2019)	To review current services and policies aimed to consolidate current research and practices in regard to	Conceptual paper (review of services and policies regarding	Asylum seekers and refugees.	Italy and Greece.	The review of current research shows that refugees have complex trauma that is aggravated by the services they receive at their reception centres and highlight the need for current refugee services and intake	Trauma, Refugees, Asylum Seekers, Mental Health.

		refugee well-being to provide direction for future policy and actions; centring around the intersection of three topics: refugees, trauma, and mental health treatment.	refugee wellbeing).			procedures to be revaluated in accordance with trauma research. The authors provide suggestions to develop refugee centres closer to major cities where they have more access to resources and can develop language and other skills to encourage growing autonomy for the refugees, in addition to creating wraparound services with refugee families, using formal and informal supports to complete the circle of support for each refugee.	
90.	Funk et al. (2012)	To highlight the health, social, economic, and human rights effects of unaddressed mental disorders in low- and middle-income countries (LMICs) and to propose effective strategies to address mental disorders and their impacts as part of an overall development strategy; by building on the findings of WHO's Report on Mental Health and Development.	Conceptual paper.	People with mental health issues.	Low- and middle-income countries.	The authors conclude that while many LMICs have identified mental health as an important issue, the finances and technical expertise to address the problem are lacking; and suggest that having mental health on the agenda of development organizations will be a critical step for overcoming the negative development consequences of mental disorders. Proposed solutions include targeted poverty alleviation programmes to break the cycle between mental illness and poverty incorporating measures specifically addressing the needs of people with mental health conditions, such as the provision of accessible and effective services and support, facilitation of education, employment opportunities and housing, and enforcement of human rights protection.	Health Policy, Mental Health Services, Development, LMICs, Human Rights, Social Equality, Inclusion, Poverty, Mental Illness, Social Care.

91.	Dopp & Lantz (2020)	To argue that the majority of evidence-based interventions focused on child and adolescent mental health are limited by their focus on individual youth (and sometimes families) using a social determinants of health framework.	Conceptual paper.	Children, adolescents and their families.	United States.	The authors conclude that these interventions are insufficient for addressing the midstream- and upstream/macro-level determinants of mental health in society by illustrating through four examples from youth mental health and related services, in which midstream and upstream interventions—i.e., at the community and public policy levels—need to be prioritized along with downstream treatments to improve population mental health and reduce social inequalities in mental health outcomes	Social Determinants of Health · Child Mental Health · Evidence-Based Interventions · Public Policy · Prevention
92.	Kamody et al. (2022)	To provide an up-to-date review of the literature on the use of medical-legal partnerships (MLPs) to address social determinants of health (SDH) needs as a means to reduce health disparities; and to make recommendations for the implementation of MLPs within paediatric psychologists' clinical work, research, and training.	Conceptual paper.	Psychologists.	Not specified.	The authors concluded that MLPs provide an opportunity for psychologists to broaden their interdisciplinary collaborations, to meet the social and legal needs of their patients more appropriately, to assist in reducing inequities among underserved paediatric populations. Recommendations include the systematic incorporation of MLPs into paediatric psychology training to increase the utilization of these services moving forward.	Health Disparities, Interdisciplinary Care, Medical-Legal Partnerships, Paediatric Psychology, Social Determinants of Health

93.	de Fátima Guareschi et al. (2010)	To discuss the effects of public policies on the constitution of the subject, from problematizations in the field of Social Psychology based on philosophy of Michel Foucault.	Conceptual paper.	Portuguese citizens.	Portugal.	The authors consider public policies as an action of the State towards the investment of the life of the population, aligning with the Brazilian CF of 1988, representing the ideal of struggle towards the guarantee of civil, political, social, economic and cultural rights of the citizen. Based on Foucault's notion of biopolitics and the construction of the Modern State, the authors explore the crossings that occur in the relationship between the State and Neoliberalism and highlight the emergence of the homo œconomicus. Authors seek to show that this dynamic establishes a conflict between the rights of the subject and the interests of the economic market, and that this relationship affects the ways of being and living of the subjects; ultimately shaping the current practices of Social Psychology.	Social Psychology, Public Policies and Subjects of Rights
94.	Davies et al. (2010)	To highlight the role of research and practice psychologists in influencing public attitudes towards poverty and shaping policy through active engagement in the policy-making process.	Conceptual paper.	Children.	New Zealand.	The authors conclude that while concerns with child poverty is partly heightened because children are dependent so society takes a broader 'duty of care' attitude - looking over the shoulders of parents and partly because adequate investment in their development is needed for the longer term good of themselves and the wider society, this viewpoint has yet to be fully accepted and institutionalised, and certainly needs to be further enhanced. The authors propose that Psychologists - including clinical,	Child Poverty | Psychologist Role | Public Attitudes | Policy Making | Poverty Prevention | Child Welfare | Policy Making | Poverty | Prevention | Public Opinion |

						forensic, developmental and educational psychologists - have important roles to play in promoting this discourse in the media and more directly with policy makers, which may assist to put genuine strategies to child poverty on the political agenda, and thereby deal with a root cause of some of New Zealand's social problems. While the costs to reduce child poverty may well be high short-term, the costs of inaction for New Zealand's children and grandchildren would likely be even higher.	Psychologists | Only Child | Child
95.	Alfaro & Martin (2015)	To review a body of literature regarding the link between academic production and the response to social problems, emphasizing the deliberative politics approach, with a description of stakeholders 'confluent and conflicting interpretations as a factor of change for public policies; and to review the literature regarding the complexity of	Conceptual paper.	Psychologists.	Not specified.	The analysis suggests that both from the field of public policy and community psychology, the main currents of analysis have a vertical perspective, which understands the use of social research as a simple knowledge transfer which oversimplifies social research as a one-way transfer of knowledge. This article highlights challenges in the relationship between community psychology and public policies and includes recommendations for adopting a "conceptualizing participant" role, which may enable community psychology to contribute from within and outside policy formulation are provided. The importance of networks, policy communities, participatory processes effective communication, understanding the policy process, and creating dialogue spaces	Community Psychology, Public Politics, Knowledge Transfer, Deliberative Public Policies, Community Intervention

		knowledge transfer from community psychology to public policy.				to bridge the gap between scientists and policy-makers in this complex dialogue.	
96.	Toquero (2021)	To call on the need for the provision of mental health services for people with disabilities, and for the governments to have a rights-based disability lens in their policy decision-making relative to emergency health response and recovery health plans.	Conceptual paper.	People with disabilities.	Philippines.	The authors highlight some of the health barriers to people with disabilities in the Philippines who continue to confront scientific, social, and systemic barriers to healthcare. The article includes recommendations for disability-inclusive emergency health response and recovery health plans in legislative policies which can leverage them to access quality healthcare in the post-pandemic period; in addition, the implementation of the Mental Health Act in the Philippines should be strengthened as the disability-inclusive legislative policies and health services should protect the rights, strengthen recovery, and fulfil the health-care needs of people with disabilities in the post-pandemic world.	Covid-19, Mental Health, Disability, Emergency Preparedness Planning, Legislation
97.	Perucchi et al. (2011)	To call a discussion about the inclusion of psychology in public policy relating to HIV/AIDS; and question what can be done to promote a reflection on the part of (the) psychologists (as) about their practices, allowing for	Conceptual paper (non-systematic literature review).	People with HIV/AIDS.	Portugal.	The authors demonstrate that psychology can play a relevant role in the development and implementation of public policies on HIV/AIDS and act beyond a healthcare practice that contributes to the promotion of human rights and citizenship. Psychology currently already has important tools to consolidate its critical and political position, especially with regard to AIDS; and the insertion of Psychology in the theme of AIDS, both in the scope of practical action	Psychology, Public Policy, Aids

		new perspectives and interventions.				and in the construction of scientific knowledge, corroborating the need for a reflection on the perspective destined to certain segments and the importance of (re) thinking about the "production of subjects" based on public policies, guidelines and actions developed; with a focus on subjectivities, allowing space for questioning and critical analysis of government strategies that produce and are produced by society.	
98.	Vodanovich & Piotrowski (2011)	To reviews key cases and legislation in the recent past, with an emphasis in the area of personnel psychology: a) age discrimination, b) retaliation) disability, d) employment testing, and e) pay inequity.	Conceptual paper.	Industrial and Organisational Psychologists.	United States.	The authors conclude that while prior research has indicated that formal education and training in legal issues has not kept pace with developments in contemporary employment settings, the legal issues have become a prominent aspect in the practice of organizational consulting. This article provides a brief overview of supercritical legal topics that continue to impact the field of I-O psychology. In addition, the authors hope that this information will provide practitioners with the impetus for further leaming and training to increase their level of professional competence.	Legislation | Personnel Psychology | Age Discrimination | Retaliation | Training | Age Discrimination | Industrial and Organizational Psychology | Legislative Processes | Legal Decisions | Training | Self Psychology
99.	Johnson (2010)	To explore the psychological impact felt by transgender individuals when ongoing	Conceptual paper.	Transgender individuals.	United States.	The authors highlight the long history of discrimination towards transgender persons and expose the society's three-pronged assault on the transgender community: 1. no one is there to protect them from abuse, 2.	Transgender Inequality | Mental Disorder | Psychological Impact |

		discrimination and violence against them is too often misunderstood, ignored, and perhaps perpetuated by legislatures and the judiciary.				no one is there to punish those who victimize them, and 3.no one is there to take care of them after they have been victimized. It is difficult for the transgender community to gain acceptance within society when government-sanctioned policies relegate these individuals to the status of nonhumans. Recommendations include changing judicial attitudes towards this community, enacting transgender-specific antidiscrimination and hate crime legislation at all levels of government to increase acceptance; and to shift society's focus away from a binary man-woman system towards viewing gender on a sliding scale.	Antidiscrimination Legislation | Judicial Unwillingness | Discrimination | Judges | Laws | Social Equality | Transgender | Socioeconomic Factors
100.	Benelli (2016)	To present a critical overview of the historical journey and the process of institutionalization of social assistance as a public policy; and to address the discourse about the social risk involved in it and in the national policy for children and adolescents for the analysis of the normalizing possibilities of	Conceptual paper.	Children and adolescents; psychologists.	Portugal.	The authors conclude that for Social Assistance to be effectively addressing social problems, it must be ethically guided by the citizen subject of rights. The historical transformations reveal a care system with predominantly preventive, corrective, repressive and resocializing characteristics, with institutional links. Therefore, the authors stress that it is important to list the practices carried out and the discourses enunciated by different sets of social actors throughout history, in a systematic way, as radicalizing the analysis allows more visibility to what is intended to be overcome, as the challenge is to construct psychosocial care practices that can	Social Assistance, Institutional Evaluation, Public Policy, Social Psychology

		Psychology and Social Care.				overcome both disciplinary and control practices in the present day.	
101.	García-Vázquez et al. (2020)	To aid in finding solutions to how the school psychology community can serve the diverse society in which we live without explicit and intentional education and growth in this area.	Conceptual paper.	Educational psychologists.	United States.	The authors emphasise that school psychologists have an ethical responsibility to engage in social justice and antiracist action. Graduate education programs and school psychology organisations have an important role in shaping future generations of school psychologists to lead the mental health, educational and research, and advocacy initiatives that promote equity for school personnel, students, families and communities they serve. The authors conclude this may be only possible if our field acknowledges, evaluates, and works to reconstruct existing systems, structures, and policies that lead to inequitable outcomes for some groups and not others. Action plan and commitments are included in the article.	Antiracism, Diversity, Equity, Policy, Action, School Psychology
102.	Migacheva (2015)	To explore areas within human rights policy, which could benefit from psychological knowledge and inquiry.	Conceptual paper.	Not specified.	Not specified.	The author discusses the different ways in which U.S. public policy encompasses human rights; and then examines how social psychology may help address some of the challenges that arise in human rights policy today. The author concludes that despite limited involvement, psychologists can close the gap between psychological knowledge and human rights policy by furthering research, and staying informed, in addition to actively engaging with the policy makers.	Social Psychology | Human Rights | Public Policy | Government Policy Making | Humanities | Humanism | Humans | Self Psychology

103.	Yadegarfard & Bahramabadian (2014)	To investigate the necessity of revising the Ethics Code of the Psychology and Counselling Organization of the Islamic Republic of Iran (PCOIRI), with respect to people's rights and dignity and to avoid unfair discriminations toward sexual orientation and gender identity.	Conceptual paper.	Psychologists; LGBTQ+ clients.	Iran.	The authors stress the need for clear and specific guidelines and a professional ethics code developed from more appropriate reference points than legal and religious sources. They argue that confused diagnoses, wrong decision making, unethical practice, and the subsequent harm caused to LGBTQ+ clients, results from the lack of a clear code and relevant guidelines and remind us that that certain conditions in Iran (religious perspectives, laws, cultural biases, misunderstandings, a lack of local academic and scientific resources, and Western conspiracy theories) lead professional psychologists and researchers to avoid researching sexual minorities and gender identity, which negatively impacts the Ethics Code of the PCOIRI. Iranian and Western researchers have identified four primary sources to which psychologists and counsellors can refer when faced with ethical dilemmas: developmental psychology, professional ethics codes, philosophy of psychology literature, and available research and compilations on professional ethics.	Psychology And Counselling Organization of I. R. Iran's (Pcoiri) Ethics Code, Sexual Orientation and Gender Identity, Religion, Law, Cultural Biases, Western Conspiracy
104.	Hatzenbuehler (2010)	To provide a synthesis of the empirical literature on the relationship between social policies and adverse mental health	Conceptual paper (synthesis of the empirical literature).	LGB populations.	Not specified.	The authors link between policies and mental health disparities by three mechanisms: decreased access to resources, increased exposure to minority stressors, and elevated psychological risk factors. Psychologists are uniquely suited to address	Social Policies | Lesbian | Gay | Bisexual | Mental Health Outcomes | Risk Factors |

		outcomes in LGB populations.				mechanisms that can explain how social/structural stressors, including policies, ultimately create disparities in psychiatric morbidity; and psychologists can play an important role alleviating the disproportionate burden of adverse mental health outcomes in LGB populations; in partnerships with social policy makers, to promote changes that reduce mental health disparities by identifying targets for preventive interventions with individuals across multiple settings and levels.	Government Policy Making | Male Homosexuality | Mental Health | Health Disparities | Bisexuality | Lesbianism | Public Policy
105.	Pettigrew (2011)	To highlight the historical and influential contributions of the Society for the Psychological Study of Social Issues (SPSSI)'s and its influence on racial research on public policy.	Conceptual paper.	Psychologists.	United States.	The review highlights the SPSSI's significant impact on public policy, particularly influencing key U.S. Supreme Court decisions on racial matters. However, the influence on consulting on these matters is less clear as some members, backed by SPSSI research, have advised political figures, while other members of SPSSI oppose racial change. The organization's policy influence fails the founders' visions, often neglecting the perspectives of social psychology. Regardless of abundant resources, SPSSI and social psychologists face challenges in making their insights widely accepted, emphasizing the ongoing importance of social psychology's contributions in shaping behaviour and understanding subjective evaluations of reality.	Racial Research | Society for The Psychological Study of Social Issues | Public Policy | Government Policy Making | Professional Organizations | Racism | Social Issue

106.	Sanchez-Mazas (2015)	To examine the case of Switzerland, where the suppression of social assistance for rejected asylum seekers is intended to support asylum policies; including a discussion of some implications of the Swiss system in the realm of education.	Conceptual paper.	Asylum seekers.	Switzerland.	The authors report findings from a Swiss field study which reveals how, the asylum policy induces institutional practices that prevent, rather than promote, failed asylum seekers to leave the territory; which leads to the construction of an invisible population and the creation of a category of people who depend on and are under the control of the very authority that tries to deport them turning those who remain in the country in conditions of total deprivation of rights and under the threat of being arrested or subject to forced departure for illegal stay as official outlaws. Discussion regarding some implications of the Swiss system in the realm of education, suggests that the institutional constraints imposed by the asylum policy jeopardize the implementation of the recognized unconditional right of children to education.	Failed Asylum Seekers, Social Assistance, Voluntary Return, Asylum Policy, Education, Children's Rights, Learned Helplessness, Psychological Reactance
107.	Cosgrove et al. (2020)	To present a discussion of how a rights-based approach can promote the voice and participation of people with lived experience into the movement for global mental health (MGMH).	Conceptual paper.	People with lived experience into the movement for global mental health.	Global.	The authors argue that a human rights framework can be enhanced by incorporating the conceptual approaches of critical inquiry and community mental health and discuss how rights-based approaches and service-user activism can productively reconfigure Western psychiatric conceptualizations of distress and provide both a moral and empirical justification for a paradigm shift within the MGMH. In conclusion it is suggested that as well-intentioned as the currently urged	Global Mental Health · Community Mental Health · Rights-Based Approach · Community Psychiatry

						technological solutions are, they are not robust enough to animate our ethical imagination, address the global burden of obstacles, or attend to the 'cultural situatedness' of distress; with many clinicians demonstrating the need for new discourses and policies that promote equality (not just treatment) as a dominant trope.	
108.	Hennes & Dang (2021)	To present examples from case law and empirical legal studies illustrate how precedent may impede social justice in ways that are predictable from psychological theory; highlighting particular barriers to justice which is disproportionately encountered by members of historically disadvantaged groups.	Conceptual paper.	Historically disadvantaged groups, judges, and lay people.	Not specified.	The authors conclude that the operation of legal precedent can, in at least some cases, perpetuate injustice, and such costs disproportionally impact historically disadvantaged group members. Encouragement to scholars and policy-makers for continuing to rigorously examine the legal and extra-legal factors that perpetuate inequities in the courtroom.	Legal Decision-Making, Precedent, Status Quo Bias, System Justification, Information Processing, Motivated Reasoning, Heuristics, Racial Disparity, Social Justice, Psychology and Law
109.	McVeigh & MacLachlan (2022)	To consider the role of psychological governance in responding to COVID-19 pandemic; and to examine the role of several	Conceptual paper.	Not specified.	Not specified.	Psychological governance and a macropsychology perspective are crucial to supporting pandemic preparedness, coping, and recovery at the population level.	Covid-19, Pandemic, Psychological Governance, Behavior Change, Policy

		macropsychological factors in the pandemic, including heroism, trust in government, culture, equitable access, and human rights.					
110.	Cook & Roesch (2012)	To review whether there is empirical support for the rationale of the proposed justice policy in Canada and the related assumption that this policy will reduce crime and better protect the public.	Conceptual paper.	Perpetrators of crime; public safety.	Canada.	The authors conclude that 'tough on crime' policies are not supported by the scientific literature as the existing literature clearly suggests that: (1) crime is not on the increase in Canada, (2) it is unlikely that the reforms will lower crime rates, and (3) there is a large financial and human cost of the recent and proposed criminal justice policies. Therefore, the authors would argue for evidenced-based justice policy; specifically, that early intervention, prevention, and rehabilitation are more beneficial in reducing crime in the long-term and more cost-effective.	Justice Policy, Reforms, Canada, Tough on Crime, Public Safety
111.	De Freitas et al. (2014)	To explore and summarize lessons learnt from three European countries (The Netherlands, Norway and Spain) experiences with migrant user involvement; including: community mobilization,	Conceptual paper.	Migrants.	Netherlands; Norway; and Spain.	The authors stress the need to open up health participatory for members of disadvantaged minority groups and to include them in decision-making processes, as highlighted by several policy documents and studies. The authors suggest strategies that can be employed in the pursuit of that goal while acknowledging that there is not a single, encompassing solution to the problem of inclusiveness in health participatory governance by offering several insights that	User Involvement, Health Policy, Health Equity, Migration, Community Psychology

		sociopolitical development, creation of community alliances and coalitions, by employing a community psychology approach.				can transform policy-making into a more inclusive process.	
112.	Javakhishvili et al. (2020)	To outline the European Society for Traumatic Stress Studies (ESTSS) strategy to address the mental health issues related to COVID-19 with focus on (1) trauma-informed policies, (2) capacity building, (3) collaborative research and (4) knowledge-exchange.	Letter to the Editor.	Not specified.	Europe.	The authors suggest that Europe faces major challenges which stem from the COVID-19 pandemic, and protecting public mental health is one of these challenges as evidence suggests there may be an increase in mental health problems and psychotrauma-related reactions and conditions among affected populations. Suggestions to minimize these severe consequences include putting in place trauma-informed policies, strategies, and interventions as well as promoting evidence-based methods of trauma-specific care, tailored to the new circum-stances. Commitments to contribute to these endeavours and in the spectrum of activities outlined above are given by the ESTSS.	COVID-19, Europe, ESTSS, Public Mental Health, Psychotraumatology, Trauma-Informed, Trauma-Specific, Prevention
113.	Hall & Yee (2012)	To consider U.S. mental health policies that have contributed to the general neglect of people of colour, as well as beliefs and attitudes specific to the neglect of Asian	Conceptual paper.	Asian Americans.	United States.	The authors propose that while many of the public policies that were most friendly to the needs of Asian Americans and other populations of colour, became law in the 1960s, many of the advances were undermined from the 1970s. However, the authors highlight cause for optimisms due to the 2010 Patient Protection and Affordable	Asian Americans, Mental Health Policy, Acculturative Stress, Discrimination, Ethnic Identity

		Americans' mental health needs.				Care Act. The authors call for urgent research and development for Asian Americans; and stress that political efforts must be made to get Asian Americans into positions of leadership and power in which they can make decisions about mental health policy priorities, as the mental health needs of Asian Americans are unlikely to be-come a national priority unless those in power are concerned about these needs.	
114.	Muniz Neto et al. (2014)	To analyse the category 'poor adolescent' as a whole population to be attended by Brazilian government from the standpoint of Michel Foucault's genealogical perspective; to link analyses concerning the history of paternalistic thinking on 'poor adolescent' in the Brazilian context and how this group became the target of a set of State practices and public policies.	Conceptual paper.	Adolescents.	Brazil.	The authors highlight hyposufficiency's significant role in societal discourse, prompting active governmental strategies directed at historically marginalized individuals. The ECA and related legal codes in Brazil intricately address the specific needs of impoverished teenagers within the human rights framework. Integrating hyposufficiency into the consumer market, the ECA fosters autonomy through inclusive education and public policies. Despite contemporary terminology emphasizing adolescents as rights-bearers, the persistent focus on socioeconomically disadvantaged youth challenges prevailing social assistance notions. This nuanced perspective reveals a complex interplay and hybridization between historical and contemporary paradigms endorsed by the ECA.	Social Psychology, Adolescence, Child and Adolescent Statute.

115.	Lessard & Lawrence (2022)	To summarise research regarding the extent of weight-based disparities in youth mental health and describes the social underpinnings of these disparities across contexts.	Conceptual paper.	Youth with overweight or obesity.	United States.	The authors find that existing research on youth with higher body weight has emphasized individual differences relative to their peers with "normal" weight, and societal stigma as an underlying driver of such differences. Strong support from youth and adults for policy actions towards concrete policy actions to minimize youths 'exposure to and experiences of weight stigma is emphasized. The authors suggest that the policy makers have an important role to play in enacting targeted approaches to address entrenched weight stigma by understanding that disparate emotional and psychological distress among this growing population stems not from high weight itself, but from the unjust social mistreatment.	Weight Stigma, Youth, Mental Health, Policy, Schools.
116.	Leslie & Mancheste r (2011)	To provide comments on an article which identifies several steps for closing the gap between the work-family practices identified by researchers as effective and the practices currently used in organizations by arguing that another key change in	Conceptual paper. Comments on an article by Ellen Ernst Kossek, Boris B. Baltes and Russell A. Matthews.	Employees.	Not specified.	The authors argue that de-gendering work and family conflict is a critical next step for increasing the prevalence and success of work – family initiatives, with some organizations moving toward this end by replacing the term ‘‘work – family policies’ with ‘‘work – life policies’’. The authors urge practitioners and academics to take steps to further reduce the assumption that work – family conflict is a women’s issue and mitigate perceptions that only a subset of employees benefit from such policies. Suggestions include moving oversight of	Work Family Research | Organizations | Policy Making | Industrial & Organizational Psychology | Family Work Relationship | Organizations | Policy Making

		the dialogue surrounding work-family conflict is needed before organizations are likely to view work-family initiatives as a strategic imperative.				work – family initiatives from diversity and inclusion departments to organizational effectiveness departments as a way to signal that reduced work – family conflict can benefit all employees, regard-less of gender; and seeing de-gender work – family conflict as a mechanism for combating gender-based discrimination, not denying its existence.	
117.	de la Peña et al. (2019)	To provide an overview of the historical and socio-political context of family separation policies in the US; to describe how zero tolerance policy (ZTP) was implemented in actuality; to offer a review of the literature on the impact of family separation on children and parents in diverse contexts; to describe direct clinical experiences with these children and parents receiving services at the Terra Firma program in the Bronx community in New York.	Conceptual paper.	Children and families experiencing separation.	United States.	The authors provide plentiful historical, scholarly, empirical, and direct case study evidence of both the unjustified, race-based character of the ZTP, as well as evidence of damaging, possible life-long effect it is having on children and parents separated at the border. The authors also outline important recommendations for policy makers, service providers, and the community as a whole, which include: avoiding separations of child from parent, informed procedures and trained staff when separations has been necessary, prompt reunifications, providing support services including mental health support and legal counsel to children and families throughout the immigration paths.	Parent-Child Separation, Children, Refugee, Immigration, Attachment., Unaccompanied, Trauma, Mental Health, Zero Tolerance Policy

118.	Liu & Peng (2012)	To examine challenges and contributions of cultural psychology to empirical legal studies.	Conceptual paper.	Eastern Asian and North American cultures.	East Asia and North America.	Cultural differences could impact people's understanding, construction and use of laws in basic ways. It is important for both psychology and law scholars to examine the ways in which the law's substantive and procedural standards are applied across different cultural populations.	Empirical Legal Science, Cultural Psychology, Value Orientations, Moral Judgments, Cognitive Styles.
