# Supplementary material for: Macropsychology: A Systematic Scoping Review of the Psychology Literature on Public Policy and Law
Source: Behav Sci (Basel). 2025 Mar 12;15(3):350. doi: 10.3390/bs15030350 (PMC11939743; doi:10.3390/bs15030350)
Supplement: Supplementary file 1 [file behavsci-15-00350-s001.zip › Supplementary File S3_Electronic Search Strategy.pdf]

Supplementary File S3

Electronic Search Strategy and Results

(Search conducted on 13th June 2022)

**Host**	**Data Base**	**Scope**	**Coverage**	**Inclusion**	**Results**
EBSCOhost	APA PsycINFO	Psychology	International	Search 1 and Search 2	7619 (29 duplicates deleted, 7590 publications remaining)

**Search Nr.**	**Search Engine**	**Search Terms Within Field**	**Search Mode**	**Limiters**	**Results**
1	EBSCOhost: APA PsycInfo	Terms 1 AND 2 within Keywords	Find All My Search Terms	Peer reviewed, 2010 onwards	**7601 (imported citations)**
2	EBSCOhost: APA PsycInfo	Terms 3 within Keywords	Find All My Search Terms	Peer reviewed, 2010 onwards	**18 (imported citations)**
